# The CXCL12/CXCR4 chemokine ligand/receptor axis in cardiovascular disease

**DOI:** 10.3389/fphys.2014.00212

**Published:** 2014-06-11

**Authors:** Yvonne Döring, Lukas Pawig, Christian Weber, Heidi Noels

**Affiliations:** ^1^Institute for Cardiovascular Prevention (IPEK), Ludwig-Maximilians-UniversityMunich, Germany; ^2^Institute for Molecular Cardiovascular Research, RWTH Aachen UniversityAachen, Germany; ^3^German Centre for Cardiovascular Research (DZHK), Partner Site Munich Heart AllianceMunich, Germany; ^4^Cardiovascular Research Institute Maastricht, University of MaastrichtMaastricht, Netherlands

**Keywords:** CXCR4, CXCL12, CXCR7, MIF, cardiovascular disease, atherosclerosis, restenosis, myocardial infarction

## Abstract

The chemokine receptor CXCR4 and its ligand CXCL12 play an important homeostatic function by mediating the homing of progenitor cells in the bone marrow and regulating their mobilization into peripheral tissues upon injury or stress. Although the CXCL12/CXCR4 interaction has long been regarded as a monogamous relation, the identification of the pro-inflammatory chemokine macrophage migration inhibitory factor (MIF) as an important second ligand for CXCR4, and of CXCR7 as an alternative receptor for CXCL12, has undermined this interpretation and has considerably complicated the understanding of CXCL12/CXCR4 signaling and associated biological functions. This review aims to provide insight into the current concept of the CXCL12/CXCR4 axis in myocardial infarction (MI) and its underlying pathologies such as atherosclerosis and injury-induced vascular restenosis. It will discuss main findings from *in vitro* studies, animal experiments and large-scale genome-wide association studies. The importance of the CXCL12/CXCR4 axis in progenitor cell homing and mobilization will be addressed, as will be the function of CXCR4 in different cell types involved in atherosclerosis. Finally, a potential translation of current knowledge on CXCR4 into future therapeutical application will be discussed.

## Introduction

Chemokines are small 8-12 kDa cytokines that mediate cell chemotaxis and arrest by binding to their respective receptors on the cell surface (Blanchet et al., 2012) (Box 1). The chemokine receptor CXCR4 and its ligand CXCL12, also known as stromal cell-derived factor 1 (SDF-1), are attractive therapeutic targets in the treatment of cancer, as they support migration, proliferation, and survival of cancer cells (Teicher and Fricker, 2010; Domanska et al., 2013). Also, CXCR4 is intensively studied in different autoimmune diseases, including rheumatoid arthritis, systemic lupus erythematosus, and autoimmune disorders of the central nerve system as multiple sclerosis, for its involvement in leukocyte chemotaxis in specific inflammatory conditions (Debnath et al., 2013; Domanska et al., 2013). Furthermore, the CXCL12/CXCR4 axis plays a crucial role in the homing of stem and progenitor cells in the bone marrow and controls their mobilization into peripheral blood and tissues in homeostatic conditions as well as after tissue injury or stress. Small molecule CXCR4 inhibitors are intensively being studied as mobilizers of hematopoietic stem cells for transplantation therapy of patients with specific types of cancer (Debnath et al., 2013) (Box 2). Upregulation of CXCL12 in hypoxic conditions with subsequent mobilization of CXCR4-positive stem and progenitor cells (Ceradini et al., 2004) has prompted researchers to explore the role and therapeutic value of progenitor cells and the CXCL12/CXCR4 axis in diverse models of ischemic injury, including in heart, kidney, lung, and brain. In addition, CXCL12/CXCR4-mediated mobilization of progenitor cells has also been intensively investigated in models of vascular injury-induced restenosis as observed after e.g., organ transplantation, balloon angioplasty, or stent implantation (Schober et al., 2006), as will be discussed in more detail later. Also, CXCR4 acts as an important coreceptor for human immunodeficiency virus (HIV) facilitating its entry in host CD4^+^ T-cells. In fact, the low-molecular weight CXCR4 antagonist AMD3100, the prototype of a group of so-called bicyclams, was originally identified in 1994 as a highly potent inhibitor of HIV replication in human T-cells. Only 3 years later, it was unraveled that blockade of CXCR4 was the underlying mechanism of the HIV inhibitory function of AMD3100 (De Clercq, 2000; Debnath et al., 2013). Taken together, it is not surprising that the CXCL12/CXCR4 axis is one of the most studied chemokine ligand/receptor axes in a diversity of pathological disorders.

Box 1 **Chemokines.**

Chemokines constitute the largest family of cytokines and are defined as small molecules that trigger chemotaxis of cells along a concentration gradient. They are generally important in cell signaling (Bachelerie et al., 2014). Although chemokines are a large family, they all have the same basic structure, known as the chemokine fold, which consists of a short N-terminal region, an extended N-loop region, followed by three β-strands and one α-helix (Rajagopalan and Rajarathnam, 2006). To date, chemokines have been classified into four major subdivisions, being C-, CXC-, CC-, and CX3C-chemokines. This classification is based on the number and spacing of conserved N-terminal cysteine groups, which form disulfide bonds and are hence crucial for the spatial arrangement and stability of the chemokine (Rajagopalan and Rajarathnam, 2006). In addition to these classical chemokine groups, chemokine-like function (CLF) chemokines have recently been suggested to form a fifth subclass, which does not have the classical chemokine fold and N-terminal residues, but nevertheless exerts typical chemokine activities (Tillmann et al., 2013).

The chemokine receptors are classified according to the chemokines they bind and are divided into two groups, being G-protein-coupled receptors (GPCRs) and atypical chemokine receptors. GPCRs generally signal by activating G-proteins, leading to a plethora of cellular functions, whereas atypical chemokine receptors appear to shape chemokine gradients and scavenge chemokines in the context of inflammation independent of G-protein signaling (Bachelerie et al., 2014). Apart from the classical function of cell recruitment, chemokines also mediate arrest of rolling leukocytes through activation of integrins. Some chemokines, like CXCL12, also have a role in cell homeostasis, which together with chemotaxis and arrest is an important regulatory factor of atherogenesis, as discussed in detail recently (Zernecke and Weber, 2014).

Box 2 **CXCR4 Antagonists.**

AMD3100, also known as Plerixafor or Mozobil (Genzyme Corp), is the first CXCR4 antagonist that has been approved by the FDA as mobilizer of hematopoietic stem cells in combination with G-CSF in treatment of patients with non-Hodgkin's lymphoma and multiple myeloma, and many other small molecule inhibitors of CXCR4 are under investigation or in clinical trial for different pathological settings, as recently discussed in detail (Debnath et al., 2013). AMD3100 is the prototype of bis-tetraazamacrocycles (bicyclams), a class of highly potent HIV1 antagonists. Subsequent studies examining the effect of structural modifications of AMD3100 on pharmacokinetic properties led to the discovery of AMD3465, a monocyclam analog of AMD3100, in which the second cyclam ring of AMD3100 was substituted by a pyridinylmethylene group. Similarly as AMD3100, AMD3465 interferes with the binding of CXCL12 to CXCR4, thereby preventing CXCL12 to trigger CXCR4 endocytosis and CXCR4-induced intracellular signaling as calcium mobilization and MAPK activation. As an advantage, AMD3465 shows a 10-fold higher effectiveness in inhibiting CXCR4 activity compared to AMD3100, but has no FDA approval to date and hence is less used in studies (Hatse et al., 2005).

Here we will focus on the role of CXCR4 in coronary artery disease (CAD) (Box 3). This pathology is caused by atherosclerosis, a chronic inflammatory disease of the vessel wall characterized by the development of lesions through continuous and progressive infiltration and accumulation of lipids and leukocytes (Hansson and Hermansson, 2011; Weber and Noels, 2011). Restriction of the blood flow by extensive lesions or thrombus formation caused by rupture of unstable plaques is a main cause for myocardial ischemia and infarction. Interventions such as balloon angioplasty or stent implantation aim to re-open the occluded artery, but are often associated with a re-narrowing of the vessel lumen (restenosis) caused by injury-induced vascular remodeling and neointimal hyperplasia. Bypass grafting remains the *gold standard* therapy for severe, diffuse coronary artery occlusion, particularly in the elderly and patients with diabetes. In this context vein graft failure is a major problem and late vein graft failure is associated with neointimal hyperplasia and accelerated atherosclerosis. Interestingly, recent genome-wide association studies (GWAS) revealed *CXCL12* as an important candidate gene associated with CAD and myocardial infarction (MI), but the underlying mechanisms remain totally unclear (Burton et al., 2007; Samani et al., 2007; Kathiresan et al., 2009; Farouk et al., 2010; Schunkert et al., 2011) (Box 4).

Box 3 **Cardiovascular Disease.**

Cardiovascular disease, including ischemic stroke and heart attack, is a leading cause of death and morbidity worldwide. Its underlying pathology, atherosclerosis, is defined as a chronic inflammatory disease of arterial walls (Hansson and Hermansson, 2011; Weber and Noels, 2011). Atherosclerotic lesion formation is initiated by dysfunction of the endothelial layer lining the arterial wall, caused by irritative stimuli such as dyslipidemia. Upon endothelial activation, monocytes start adhering to and migrating through the endothelium. Monocyte-derived macrophages in the arterial wall take up cholesterol-rich LDL particles, leading to the formation of so-called foam cells. As the atherosclerotic lesion progresses, smooth muscle cells (SMCs) migrate from the media to the intima, resident intimal SMCs proliferate and extracellular matrix molecules such as elastin, collagen and proteoglycans are synthesized. A necrotic core made of extracellular lipids derived from necrotic and apoptotic foam cells forms in advanced plaques, along with a fibrous cap consisting of collagen and SMCs. The ultimate complications of atherosclerosis are flow-limiting stenosis and plaque rupture, the latter triggering vessel occlusion through thrombus formation.

Box 4 **Genome-wide Association Studies.**

Genome-wide association studies (GWAS) have emerged as a very powerful tool in medical research over the last decade. In association studies, the frequency of alleles or genotype-variants is compared between disease cases and controls. GWAS apply this principle to the whole genome, i.e., a dense set of single nucleotide polymorphisms (SNPs) across the whole genome is genotyped to find out the most common variation of SNP patterns in a disease of interest (Hirschhorn and Daly, 2005). This method is a comprehensive, unbiased approach to identify genes which are regulated in a disease of interest.

Since CAD is a multifactorial disease, it is a highly interesting target for GWAS. Indeed, several GWAS in the context of CAD have been performed over the last years by the Wellcome Trust Case Control Consortium, the Ottawa Heart study, the Myocardial Infarction Genetics Consortium and others (Schunkert et al., 2011; Maouche and Schunkert, 2012). The first locus that was identified and could be replicated in all CAD-related GWAS was a strong signal on chromosome 9p21 (Farouk et al., 2010). Another strong locus that has sparked particular interest is on chromosome 10q11, close to the gene encoding CXCL12 (Farouk et al., 2010).

To facilitate future research exploring the role of CXCL12 and CXCR4 in CAD, this review aims to discuss the current concept of the CXCL12/CXCR4 axis in atherosclerosis, injury-induced vascular restenosis and MI in relation to its role in progenitor cell mobilization and biological functions in atherosclerosis-relevant cell types. We will also introduce MIF as an alternative chemokine ligand for CXCR4, and CXCR7 as an additional receptor for CXCL12, to emphasize the complexity of identifying specific CXCL12- and CXCR4-associated functions through intertwining of chemokine (receptor) signaling.

## CXCR4 as a chemokine receptor for CXCL12 and MIF

### CXCR4 and its chemokine ligand CXCL12

**Figure 1:**
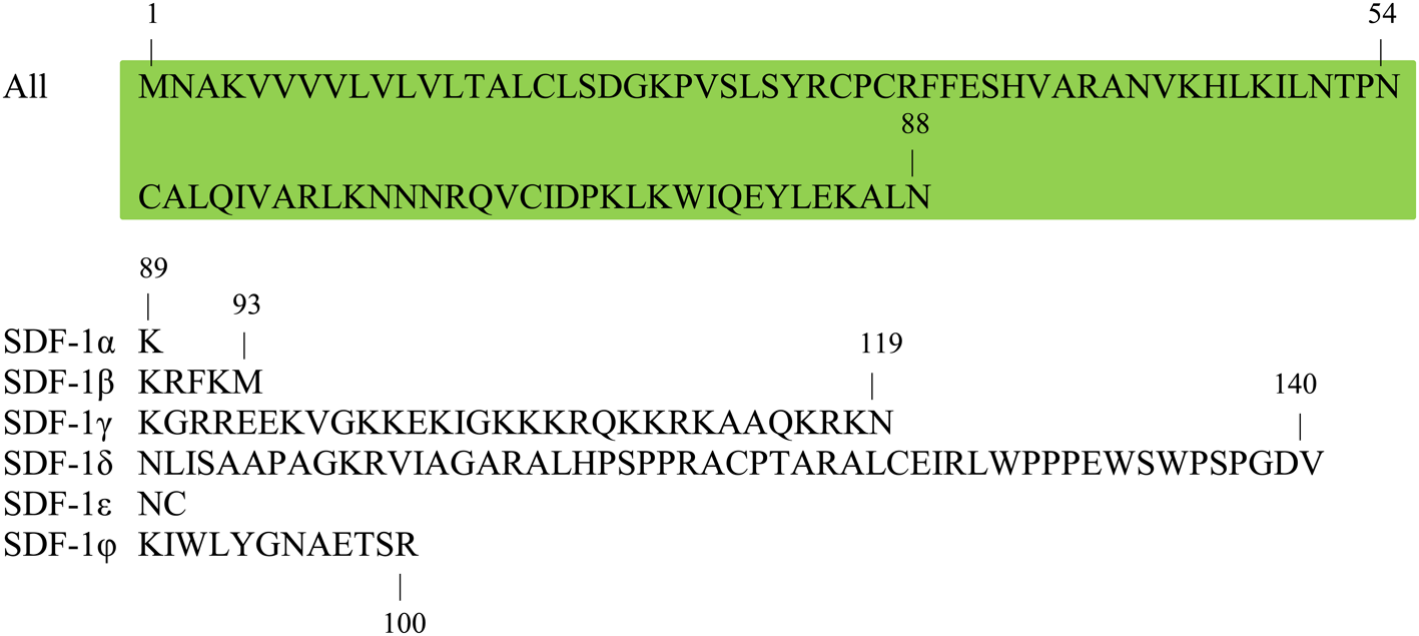
**Sequence similarities of different CXCL12/SDF-1 isoforms.** Six isoforms of CXCL12/SDF-1 have been described to date, which share a common N-terminal amino acid sequence, but a distinct C-terminus. Shown is the single letter amino acid code for all CXCL12 isoforms, with the shared N-terminal sequence highlighted in green. Indications of specific amino acid positions inform on the length of the different CXCL12 isoforms: α (89 aa), β (93 aa), γ (119 aa), δ (140 aa), ε (90 aa), and ϕ (100 aa). *aa, amino acid; SDF, stromal cell-derived factor.*

The chemokine receptor CXCR4 belongs to the family of seven-span transmembrane G-protein-coupled chemokine receptors (GPCRs). It is ubiquitously expressed and evolutionary conserved, with 89% of similarity between the human and mouse protein. In 1996 SDF-1, later called CXCL12, was identified as a ligand for CXCR4 (Bleul et al., 1996a; Oberlin et al., 1996). Similar as CXCR4, CXCL12 is highly conserved, with human and mouse showing around 92% amino acid (aa) identity for both ubiquitously expressed isoforms α (89 aa) and β (93 aa) (Shirozu et al., 1995). Except for these 2 classical isoforms, which are so far functionally indistinguishable and differ only in 4 amino acids at the C-terminal end, four additional isoforms have been identified in humans. These γ (119 aa), δ (140 aa), ε (90 aa), and ϕ (100 aa) isoforms show a more restricted expression pattern and have been much less studied. They result from differential splicing and only differ at their C-terminal region (Yu et al., 2006) (Figure 1).

Mice deficient for *Cxcl12* or *Cxcr4* die perinatally due to defects in hematopoiesis, vasculo-, cardio-, and neurogenesis (Nagasawa et al., 1996; Ma et al., 1998; Tachibana et al., 1998; Zou et al., 1998; Ara et al., 2005). The importance of the CXCL12/CXCR4 axis in embryonic development is associated with an essential role in homeostatic (progenitor) cell migration. This clarifies the classification of the CXCL12/CXCR4 axis into the “homeostatic/constitutive” chemokine ligand/receptor group, instead of the “inflammatory/inducible” group of chemokines, which groups chemokines that are upregulated during inflammation to drive immune responses (Bachelerie et al., 2014). The CXCR4 receptor and its ligand CXCL12 are mostly studied for their crucial role in the homing of (hematopoietic) progenitor cells in the bone marrow and their mobilization into the periphery in physiological and pathological conditions. Furthermore, the CXCL12/CXCR4 axis is involved in chemotaxis, cell arrest, angiogenesis, and cell survival. These functions explain not only the interest of oncologists in CXCR4, but also underlie the involvement of the CXCL12/CXCR4 axis in CAD, as will be discussed in more detail later.

**Figure 2:**
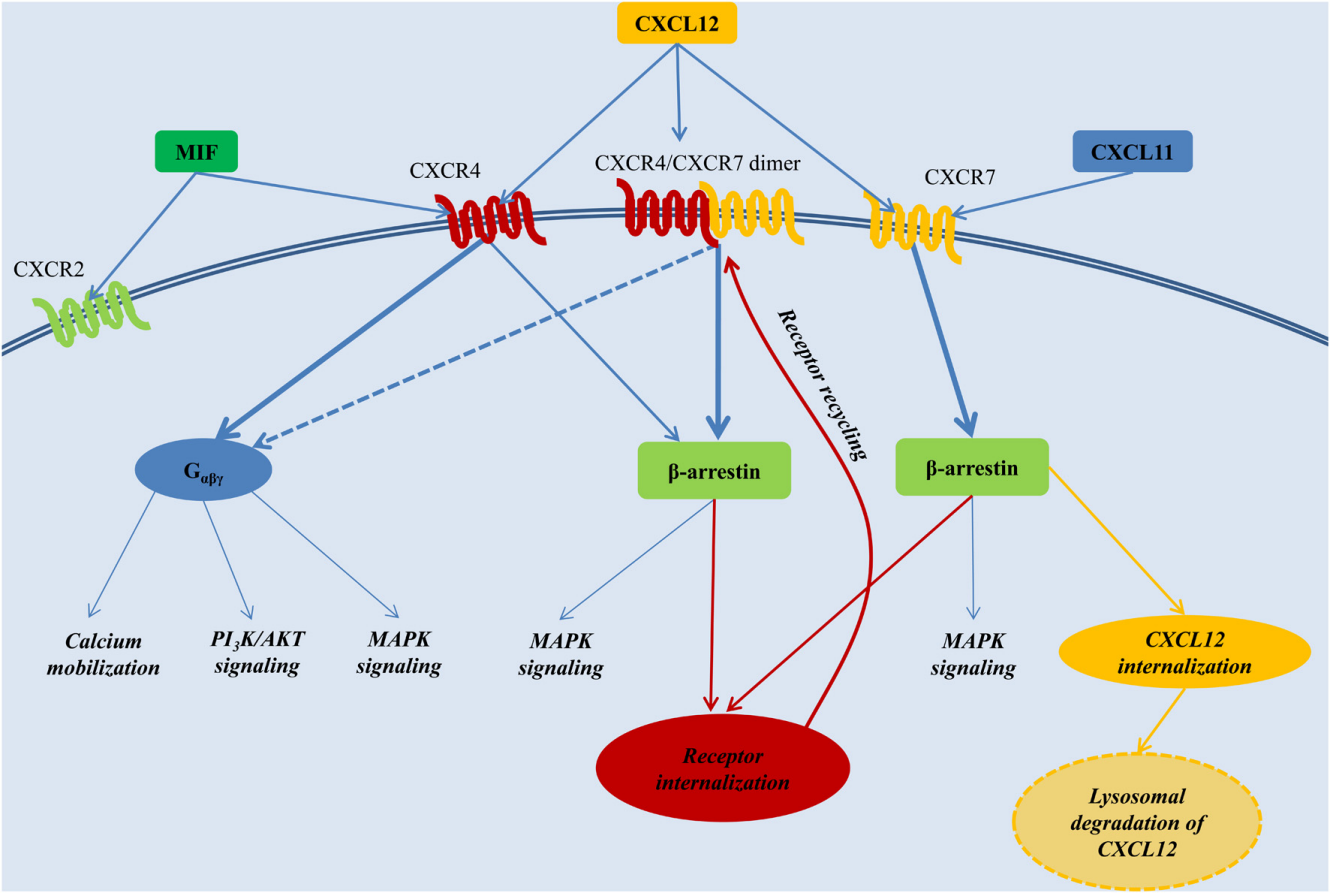
**The CXCL12 signaling network.** CXCL12 employs two distinct receptors, CXCR4 and CXCR7. CXCR4 additionally acts a receptor for MIF, whereas CXCR7 can also bind CXCL11. Generally, stimulation of CXCR4 triggers preferentially G-protein-coupled signaling, whereas activation of CXCR7 or the CXCR4/CXCR7 complex induces β-arrestin-mediated signaling. Internalization of the receptors CXCR4 and CXCR7, and subsequent recycling to the cell membrane, is also mediated through β-arrestin. Upon binding to CXCR7, CXCL12 is internalized and subjected to lysosomal degradation. AKT, PKB, Protein kinase B; MAPK, mitogen-activated protein kinase; MIF, macrophage migration inhibitory factor; PI3K, phosphatidylinositide 3-kinase; G_αβγ_, heterotrimeric G-protein consisting of the subunits α, β, and γ.

Binding of CXCL12 to CXCR4 mediates intracellular signaling through a classical heterotrimeric G-protein, composed of an Gα, Gβ, and Gγ subunit. Of the 4 general classes of Gα proteins, named Gα_s_, Gα_i_, Gα_q_, and Gα_12_, CXCR4 signaling seems mainly coupled to the Gα_i_ subunit. Receptor stimulation induces the dissociation of the heterotrimeric G-protein. The Gα_i_ monomer inhibits adenylyl cyclase activity and triggers MAPK and PI3K pathway activation, whereas the Gβγ dimer triggers intracellular calcium mobilization through the activation of phospholipase C (Teicher and Fricker, 2010). Furthermore, CXCL12 induces the recruitment of β-arrestin to CXCR4. This mediates receptor desensitization through CXCR4 endocytosis (Orsini et al., 1999), but also reduces CXCR4 coupling to Gα_i_ signaling, favoring β-arrestin-mediated MAPK activation. The latter was particularly shown upon overexpression-induced dimerization of CXCR4 with CXCR7, a chemokine receptor that will be discussed in more detail below (Decaillot et al., 2011) (Figure 2). Preferential signaling through G-proteins vs. β-arrestin is not only influenced through dimer formation of CXCR4 with CXCR7, but also by the oligomerization state of CXCL12 (Drury et al., 2011; Ray et al., 2012).

### MIF as an alternative chemokine ligand for CXCR4

The CXCL12/CXCR4 axis was long considered a monogamous relation, until in 2007 the chemokine MIF was surprisingly identified as an alternative ligand for CXCR4 (Bernhagen et al., 2007). MIF is a ubiquitously expressed and highly conserved protein of 114 aa (excluding the N-terminal methionine which is post-translationally removed), with 90% homology between human and mouse (Bernhagen et al., 1994). MIF plays an important role in cell recruitment and arrest through binding to the chemokine receptors CXCR2 and CXCR4 (Bernhagen et al., 2007). However, it cannot be classified into one of the four typical chemokine classes (C,CC,CXC,CX_3_C) due to the absence of a characteristic cysteine motif in its N-terminus, and is therefore called a chemokine-like function chemokine (Box 1). In contrast to CXCL12, MIF is secreted in response to diverse inflammatory stimuli, and has been associated with a clear pro-inflammatory and pro-atherogenic role in multiple studies of patients and animal models (Pan et al., 2004; Bernhagen et al., 2007). On the other hand, MIF can also exert protective functions, as observed after myocardial ischemia/reperfusion injury (MI/IRI) and in experimental liver fibrosis. We will address the double-edged role of MIF in myocardial ischemia below. For the involvement of MIF in chronic atherosclerosis and injury-induced restenosis, we refer to a recent review by Tillmann et al. (2013).

## CXCR7, an alternative receptor for CXCL12 heterodimerizing with CXCR4

In 2005, CXCL12 was revealed to bind a second chemokine receptor, named CXCR7 (or RDC1), with an even 10-fold higher affinity compared with CXCR4 (Balabanian et al., 2005; Burns et al., 2006). Similarly as CXCR4, CXCR7 is highly conserved between human and mouse. Its deletion in mice is perinatally lethal and associated with defective cardiovascular development (Sierro et al., 2007; Yu et al., 2011b). CXCR7 has been implicated in cell survival and adhesion (Burns et al., 2006) and can mediate CXCL12-directed T-cell chemotaxis independently from CXCR4 (Balabanian et al., 2005; Kumar et al., 2012). Binding of the chemokine ligands CXCL12 and CXCL11 (also called I-TAC) to CXCR7 enhances continuous CXCR7 internalization and delivery of the chemokine ligands to the lysosomes for degradation (Luker et al., 2010; Naumann et al., 2010). Such CXCR7-mediated regulation of available CXCL12 concentrations has associated CXCR7 with a function as decoy receptor, reducing acute CXCL12/CXCR4 signaling (Luker et al., 2010). Furthermore, study of a CXCR7 agonist recently suggested downregulation of CXCR4 protein levels by CXCR7 signaling as another negative regulatory mechanism of CXCR7 toward the CXCL12/CXCR4 axis (Uto-Konomi et al., 2013). Also, heterodimerization of CXCR7 with CXCR4 interferes with CXCR4-induced Gα_i_ protein-mediated signaling and favors β-arrestin-linked signaling (Levoye et al., 2009; Decaillot et al., 2011). On the other hand, the CXCL12 scavenging function of CXCR7 can also positively influence CXCR4-mediated migration by preventing the downregulation of CXCR4 surface expression and function through excessive CXCL12 concentrations (Sanchez-Alcaniz et al., 2011). In addition to its modulatory effect on CXCL12/CXCR4 signaling, CXCR7 is able to mediate CXCL12-induced MAPK activation independently from CXCR4 (Wang et al., 2011). Although the precise signaling mechanisms downstream of CXCR7 remain unclear, CXCR7 does not bind to or induce the activation of heterotrimeric G-proteins as typical in classical GPCR signaling, but depends on ligand-induced β-arrestin recruitment (Rajagopal et al., 2010) (Figure 2). This atypical signaling explains why CXCR7 was recently renamed atypical chemokine receptor (ACKR) 3 (Bachelerie et al., 2014).

As an important consideration, the widely used CXCR4 antagonist AMD3100 (Box 2) was recently revealed to be an allosteric agonist of CXCR7, being able to induce the recruitment of β-arrestin to CXCR7 at concentrations from 10 μM. Also, AMD3100 increased CXCL12 binding and CXCL12-triggered β-arrestin recruitment to CXCR7 (Kalatskaya et al., 2009). The CXCR4 antagonist TC14012 even showed a higher agonistic effect on CXCR7 (Gravel et al., 2010). These findings, together with the regulatory effects of CXCR7 on CXCL12/CXCR4 signaling, considerably complicate the interpretation of all studies aiming to dissect the molecular mechanisms and biological consequences of specifically CXCL12/CXCR4 signaling.

## The CXCL12/CXCR4 axis mediates homing and mobilization of progenitor cells

### Homing of progenitor cells in the bone marrow

In physiological conditions low numbers of hematopoietic stem and progenitor cells (HSPCs) constantly circulate from the bone marrow to the blood and back. The CXCL12/CXCR4 axis has been shown to play a crucial role in the homing and retention of HSPCs in the stem cell niches of the bone marrow (Mazo et al., 2011). First, CXCL12 secreted by endothelial cells of sinusoids in the bone marrow may trigger firm arrest of rolling CXCR4^+^ HSPCs through CXCR4-mediated integrin activation (Peled et al., 2000; Mazo et al., 2011). After vascular extravasation, HSPCs home into specialized bone marrow niches that provide optimal conditions for HSPC survival, self-renewal, and lineage differentiation and function (Jones and Wagers, 2008). High expression of CXCL12 by bone marrow stromal cells constitutes an important adhesive mechanism to retain CXCR4^+^ HSPCs in the bone marrow.

### Stress-induced mobilization of progenitor cells

In conditions of stress or injury, HSPCs lose their anchorage in these niches and are increasingly mobilized into the circulation (Mazo et al., 2011). Interference with CXCL12/CXCR4-mediated retention is an important mechanism underlying HSPC mobilization. For example, granulocyte colony-stimulating factor (G-CSF or CSF3), a mobilizing cytokine frequently used in the clinic, reduces CXCL12 expression by bone marrow osteoblasts through depletion of endosteal macrophages that support osteoblast function (Semerad et al., 2005; Winkler et al., 2010). Furthermore, G-CSF has been associated with proteolytic inactivation of CXCR4 on HSPCs in the bone marrow (Levesque et al., 2003), despite enhanced surface expression of CXCR4 on bone marrow cells upon G-CSF treatment (Petit et al., 2002). In addition, G-CSF-induced mobilization of HSPCs involves activity of the protease dipeptidyl-peptidase 4 (DPP4, also known as CD26), which inactivates CXCL12 through proteolysis (Christopherson et al., 2003a,b; Campbell and Broxmeyer, 2008). A similar role in CXCL12 destabilization during HSPC mobilization has been suggested for the proteases matrix metallopeptidase (MMP) 9, neutrophil elastase and cathepsin G. But although these proteases are upregulated in the bone marrow upon treatment with G-CSF (Levesque et al., 2002), combined inhibition of a broad range of metalloproteinases and neutrophil serine proteases—including MMP9, neutrophil elastase and cathepsin G—did not significantly affect G-CSF-triggered HSPC mobilization (Levesque et al., 2004).

A fourth mechanism proposed to interfere with CXCL12/CXCR4-mediated retention of HSPCs in the bone marrow is an increased plasma level of CXCL12, which may favor CXCL12-induced migration of HSPCs into the circulation over their retention in the bone marrow. For example, increased concentrations of CXCL12 in the blood due to injection of CXCL12-expressing adenovirus or stabilized methionine-CXCL12 induced the mobilization of hematopoietic cells and progenitors (Hattori et al., 2001; Moore et al., 2001). However, it has been debated whether this is indeed due to CXCL12-induced mobilization, or rather to a reduced CXCL12/CXCR4-mediated retention of these cells in the bone marrow caused by CXCL12-induced downregulation of CXCR4 on their cell surface (Bonig and Papayannopoulou, 2012). Similarly, it is still debated whether the mobilization of HSPCs by CXCR4 antagonists, as was recently discussed in detail (Rettig et al., 2012), is mostly the result of a direct blockade of CXCR4 on HSPCs in the bone marrow interfering with CXCR4-mediated homing (Karpova et al., 2013), or whether these antagonists may also mediate HSPC egress by altering the CXCL12 gradient in favor of mobilization. In context of the latter, it was shown that treatment with the CXCR4 antagonist AMD3100 induces a fast release of CXCL12 from bone marrow stroma to the circulation, which may favor CXCL12/CXCR4-mediated mobilization of HSPCs into circulation over their anchorage in bone marrow niches (Dar et al., 2011).

Clearly, the CXCL12/CXCR4 axis plays a pivotal but complex role in the homing and mobilization of progenitor cells, and an intertwinement with different other pathways even further complicates the picture. For example, AMD3100-induced progenitor cell mobilization was recently shown to require the expression of MMP9 and endothelial nitric oxide synthase (eNOS, also known as NOS3) in bone marrow-derived cells (Jujo et al., 2010, 2013). Also, CXCL12-mediated homing and mobilization of progenitor cells is modulated by e.g., fms-related tyrosine kinase 3 (FLT3) ligand (Fukuda et al., 2005), transforming growth factor (TGF) β (Basu and Broxmeyer, 2005), and CCR5 chemokine receptor ligands (Basu and Broxmeyer, 2009).

### Survival and proliferation of progenitor cells

In addition to homing and mobilization, the CXCL12/CXCR4 axis provides important survival and proliferative signals to progenitor cells (Lataillade et al., 2000; Lee et al., 2002; Broxmeyer et al., 2003a,b; Guo et al., 2005). Together, these functions underlie to a considerable extent the currently known involvement of the CXCL12/CXCR4 axis in CAD, as will be discussed in detail below.

## The CXCL12/CXCR4 axis in CAD: identifying potential functions and underlying mechanisms from *in vitro*, animal and patient observations

The involvement of CXCR4 and its ligand CXCL12 in injury-induced restenosis and MI has mostly been linked to progenitor cell recruitment. In contrast, the role of the CXCL12/CXCR4 axis in native atherosclerosis remains largely unclear, with mostly only *in vitro* studies shedding some light on the effect of CXCR4 signaling on cell type-specific functions relevant in atherogenesis. Here, we aim to provide an overview of *in vitro*, animal and patient observations that may provide insight into the (cell type-specific) effects of CXCR4 signaling on native atherosclerosis, injury-induced restenosis and MI (Table 1, Supplemental Table 1).

**Table 1:** **The CXCL12/CXCR4 axis in CAD**.

**CXCL12/CXCR4 IN MYOCARDIAL ISCHEMIA**	
***Expression***	
CXCL12 and CXCR4 expressed in cardiac myocytes, fibroblasts and cardiac ECs	Hu et al., 2007; Saxena et al., 2008
Myocardial ischemia increases CXCL12 expression	Pillarisetti and Gupta, 2001; Yamani et al., 2005; Hu et al., 2007
***Cardioprotective effects of CXCL12/CXCR4 signaling***	
Reduced infarction size and increased cardiac function upon CXCL12 delivery after MI/IRI and MI	Hu et al., 2007; Segers et al., 2007; Saxena et al., 2008
Associated with:	
Increased survival of cardiomyocytes	Hu et al., 2007; Saxena et al., 2008
Increased neo-angiogenesis in infarcted region	Saxena et al., 2008
Enhanced incorporation of progenitor cells in infarcted region	Abbott et al., 2004; Elmadbouh et al., 2007; Segers et al., 2007; Purcell et al., 2012
Cardioprotective signaling through AKT and ERK	Hu et al., 2007; Saxena et al., 2008
Enhanced VEGF expression	Saxena et al., 2008
***Cardiovascular detrimental effects of CXCR4 signaling***	
Inflammatory cell infiltration	Chen et al., 2010a; Liehn et al., 2011
Enhanced TNFα expression and cardiomyocyte apoptosis	Chen et al., 2010a
***Contrasting effects of CXCR4 antagonist AMD3100***	
Single treatment: cardioprotective after MI/IRI and MI	Jujo et al., 2010, 2013
Enhanced incorporation of progenitor cells in infarcted region	
Increased neo-vascularization	
Daily treatment: cardioprotective after MI	Proulx et al., 2007
Continuous administration: reduced cardiac function and survival after MI	Dai et al., 2010; Jujo et al., 2010
Reduced incorporation of progenitor cells in the infarcted region despite enhanced mobilization	Jujo et al., 2010
Increased proliferation of resident cardiac progenitor cells (⇒ reduced differentiation?)	Dai et al., 2010
**CXCL12/CXCR4 IN INJURY-INDUCED RESTENOSIS (WIRE INJURYOF CAROTID ARTERY)**	
***Expression***	
Vascular injury increases CXCL12 expression in plasma and vascular wall	Schober et al., 2003; Yin et al., 2010
Injury-induced CXCL12 upregulation in the vascular wall is mediated through:	
HIF1α upregulation	Karshovska et al., 2007
Apoptosis in injured artery	Zernecke et al., 2005
***Detrimental effects of CXCL12/CXCR4 signaling***	
*Apoe*^−/−^ treated with CXCL12 blocking antibody	Schober et al., 2003; Zernecke et al., 2005
Reduced neointimal lesion size	
Reduced SMC content and reduced mobilization of Lin^−^Sca1^+^ cells	
*Apoe*^−/−^ transplanted with *Cxcr4*^−/−^ bone marrow or treated locally with lentivirus expressing mutant CXCL12 (antagonist)	Zernecke et al., 2005
Reduced neointimal lesion size	
Reduced SMC content	
No effect on reendothelialization	
*Apoe*^−/−^ treated with CXCR4 antagonist AMD3465	Karshovska et al., 2008
Reduced neointimal lesion size	
Reduced SMC content and mobilization of Lin^−^Sca1^+^ cells	
Reduced neointimal proliferation	
No effect on reendothelialization	
*Apoe*^−/−^ treated with CXCR4 antagonist POL5551	Hamesch et al., 2012
Reduced neointimal lesion size	
Reduced SMC content and mobilization of Lin^−^Sca1^+^ cells	
Variable effect on macrophage content and reendothelialization depending on treatment regime	
***Protective effects of CXCL12/CXCR4 signaling***	
*Apoe*^−/−^ injected with EPCs	Hristov et al., 2007
Blocking CXCR2 or CXCR4 on EPCs reduced adhesion to injured artery	
C57BL/6 injected with EPCs	Yin et al., 2010
Enhanced reendothelialization and reduced neointimal lesion size with wild-type but not CXCR4-blocked EPCs	
C57BL/6 treated with Cxcl12 blocking antibody	Yin et al., 2010
Reduced mobilization of EPCs (Sca1^+^Flk1^+^ cells)	
Reduced reendothelialization	
No effect on neointimal lesion size	
C57BL/6 injected with Foxc2-transgenic EPCs	Li et al., 2011
Prior CXCR4 blockade on EPCs reduced their adhesion to the injured artery and reduced their protective effect on neointimal lesion size	
NRMI^nu/nu^ athymic nude mice injected with CXCR4-transgenic EPCs	Chen et al., 2010b
Enhanced reendothelialization compared to infusion of wild-type EPCs	
**CXCL12/CXCR4 IN CHRONIC ATHEROSCLEROSIS**	
***Protective effects of CXCL12/CXCR4 signaling***	
*Apoe*^−/−^ treated with endothelial apoptotic bodies (high-fat diet study)	Zernecke et al., 2009
Reduced lesion size, macrophage content and apoptosis	
Effect of apoptotic bodies reversed upon treatment with AMD3100	
***Possible detrimental effects of CXCL12/CXCR4 signaling***	
*Apoe*^−/−^ with established atherosclerosis, treated with conjugated linoleic acid (CLA)	De Gaetano et al., 2013
CLA induced lesion regression	
Associated with reduced CXCR4 expression and CXCL12-induced chemotaxis of monocytes	
*Apoe*^−/−^ treated with H_2_S donor GYY4137	Liu et al., 2013
Reduced lesion size	
Associated with reduced CXCR4 expression on macrophages	
***Studies without effects of CXCL12/CXCR4 signaling***	
*Apoe*^−/−^ treated with CXCL12 neutralizing antibody during last 4 weeks of 12-weeks high-fat diet	Bernhagen et al., 2007
No effect on lesion size	
***Contrasting effects of CXCR4 antagonists***	
*Apoe*^−/−^ treated with CXCR4 antagonist AMD3465	Zernecke et al., 2008
Enhanced mobilization of Lin^−^Sca1^+^ cells and leukocytes, predominantly neutrophils	
Increased lesion size with reduced SMC content, but increased neutrophil content	
Increased apoptosis of plaque cells	
“Reversa” mouse treated with AMD3100 (high-fat diet study)	Yao et al., 2012
Enhanced mobilization of progenitor cells, including “EPCs” (Cd11b^−^ cKit^+^ Flk1^+^ or Cd34^+^Cd133^+^Flk1^+^)	
Enhanced atherosclerosis regression after plasma lipid normalization	
**CXCL12/CXCR4 IN PARTIAL LIGATION MODEL (ACCELERATED ATHEROSCLEROSIS MODEL)**	
***Protective effects of CXCL12/CXCR4 signaling***	
*Apoe*^−/−^ with systemic CXCL12 treatment	Akhtar et al., 2013
Enhanced recruitment and incorporation of bone marrow-derived Lin^−^Sca1^+^ cells	
Enhanced lesion stability, without effect on lesion size	
Reduced macrophage content	
*Apoe*^−/−^ with local *CXCL12* siRNA treatment	Akhtar et al., 2013
Increased lesion size	
Reduced SMC content and increased macrophage content	
**HUMAN STUDIES**	
***GWAS***	
SNPs rs1746048 (risk allele: C) and rs501120 (risk allele: T) on Chr10q11.21, 80 Kb downstream of *CXCL12*	
Significantly associated with CAD and MI risk	Burton et al., 2007; Samani et al., 2007; Kathiresan et al., 2009; Schunkert et al., 2011
Although genome-wide significance could not be reached in 2 other studies	Ripatti et al., 2010; Peden et al., 2011
***CXCL12 expression***	
CXCL12 protective?	
Significantly reduced CXCL12 plasma levels in patients with angina, especially in unstable angina	Damas et al., 2002
CAD risk genotype rs501120 (T/T) significantly associated with reduced CXCL12 plasma levels	Kiechl et al., 2010
CXCL12 progressive?	
Risk alleles of rs1746048 and rs501120 significantly associated with higher CXCL12 plasma levels	Mehta et al., 2011
***CXCR4 expression***	
Patients with angina show reduced CXCR4 surface expression on peripheral blood cells	Damas et al., 2002

### CXCR4 in myocardial ischemia

#### Protective effects through cardiomyocyte protection and progenitor cell recruitment

CXCR4 and its ligand CXCL12 are expressed in cardiac myocytes and fibroblasts, and myocardial ischemia significantly upregulates CXCL12 (Pillarisetti and Gupta, 2001; Yamani et al., 2005; Hu et al., 2007). Different studies have revealed a protective role for CXCL12/CXCR4 signaling after MI and MI/IRI through survival effects on resident cardiomyocytes and recruitment of protective circulating cells. Intracardiac or intramyocardial injection of CXCL12 reduced infarction size and increased cardiac function after MI/IRI (Hu et al., 2007) and MI (Segers et al., 2007; Saxena et al., 2008), and cardioprotective effects were blocked with AMD3100 (Hu et al., 2007). CXCL12-induced cardioprotection was associated with improved survival of hypoxic myocardium and increased neo-angiogenesis, and was linked with anti-apoptotic AKT (also known as PKB = protein kinase B) and MAPK3/1 (also known as ERK1/2) signaling in cardiac myocytes and endothelial cells (Hu et al., 2007; Saxena et al., 2008). Also, delivery of CXCL12 triggered upregulation of vascular endothelial growth factor (VEGF) in the infarcted area and in cardiac endothelial cells, with VEGF an important regulator of angiogenesis and progenitor cell recruitment (Saxena et al., 2008). Furthermore, multiple studies have explored the effect of CXCL12 delivery into the myocardium through local treatment with CXCL12-overexpressing adenovirus, CXCL12-transgenic skeletal myoblasts or CXCL12-releasing hydrogels. Such exogenous CXCL12 delivery was associated with enhanced recruitment and incorporation of CXCR4^+^ stem and progenitor cells in the infarcted area (Abbott et al., 2004; Elmadbouh et al., 2007; Segers et al., 2007; Purcell et al., 2012).

Stem cell transplantation during MI seems promising to improve cardiac outcome (Sanganalmath and Bolli, 2013), but it is debated whether this is primarily mediated through direct regeneration of cardiac myocytes or through protective paracrine effects on remodeling or preservation of injured tissue (Liehn et al., 2013b). For example, transplantation of endothelial progenitor cells (EPCs) was associated with increased neovascularization and improved cardiac function after MI and MI/IRI, despite variable effects on inflammation and apoptosis (Schuh et al., 2008, 2012). Overexpression of CXCL12 in transplanted EPCs further increased angiogenesis, however without significant improvement of cardiac function (Schuh et al., 2012). In a complementary approach, transplantation of mesenchymal stem cells (MSCs), which have been described as cardiac precursors, is widely investigated as a potential therapy after MI (Hatzistergos et al., 2010; Dong et al., 2012; Liehn et al., 2013b). Overexpression of CXCL12 in transplanted MSCs improved survival of cardiomyocytes after MI, however without evidence for cardiac regeneration (Zhang et al., 2007). MSCs with transgenic CXCR4 expression displayed increased incorporation into the ischemic area, which was associated with increased angiogenesis, myogenesis and cardiac function (Zhang et al., 2008; Huang et al., 2012). *In vitro* experiments demonstrated hypoxia to increase CXCR4 expression on MSCs, and CXCR4-mediated migration of MSCs to CXCL12 was shown to require PI3K/AKT signaling (Yu et al., 2010). The CXCL12/CXCR4 axis was also at least partly responsible for the beneficial effects of VEGF-overexpressing MSCs by mediating the recruitment of cardiac stem cells to the infarcted region (Tang et al., 2011). Furthermore, cardioprotective effects of transplanted MSCs were shown to require myocardial CXCR4 expression (Dong et al., 2012).

The efficiency of AMD3100 in mobilizing progenitor cells, including EPCs, from the bone marrow was associated with enhanced accumulation of progenitor cells in the infarcted tissue, enhanced neovascularization and improved cardiac function after single AMD3100 treatment in an MI and MI/IRI model (Jujo et al., 2010, 2013). Similarly, cardioprotective effects were reported of daily AMD3100 injections after MI in rats (Proulx et al., 2007). However, two independent groups revealed a reduced cardiac outcome after MI upon chronic AMD3100 administration (Dai et al., 2010; Jujo et al., 2010). This was either associated with a reduced incorporation of progenitor cells in the infarcted region despite enhanced mobilization (Jujo et al., 2010), or with an increased proliferation of resident cardiac progenitor cells. This latter observation raised the suggestion whether increased proliferation may be linked to reduced differentiation, so whether cardioprotective CXCR4 signaling may be required to direct cardiac progenitors to cardiac commitment to ensure their participation in repair of injured myocardium (Dai et al., 2010).

Interestingly, platelet-surface binding of CXCL12, which correlated with platelet activation, was significantly increased in patients with acute coronary syndrome (ACS) compared to patients with stable angina pectoris, and correlated with the number of circulating hematopoietic progenitor cells (Stellos et al., 2009). Similarly, surface expression of CXCR7 but not CXCR4 was found to be significantly enhanced on platelets from patients with ACS compared to subjects with stable CAD (Rath et al., 2014). These data may be supported by recent findings that CXCL12 upregulates CXCR7 surface availability on platelets (Chatterjee et al., 2014), as will be discussed in more detail later. Of note, platelet CXCR7 surface expression levels above average in patients with ACS positively correlated with an increase in the left ventricular ejection fraction as a measure of recovery after MI, suggesting a beneficial effect of CXCL12/CXCR7 signaling on functional recovery in ACS patients (Rath et al., 2014).

#### Double-edged role of CXCR4 in the ischemic heart

Despite cardioprotective functions of the CXCL12/CXCR4 axis in the ischemic heart, *Cxcr4*-heterozygosity in mice reduced infarct size after MI, however without affecting cardiac function. This was explained by a counterbalance of on the one hand reduced neovascularization, and on the other hand reduced inflammation with less neutrophils and a preferential recruitment of Gr1^low^ over inflammatory Gr1^high^ monocytes (Liehn et al., 2011). Likewise, adenovirus-mediated overexpression of CXCR4 in the heart increased infarct size and reduced cardiac function. This was associated with an enhanced recruitment of inflammatory cells, enhanced tumor necrosis factor (TNF) α expression and increased apoptosis of cardiomyocytes (Chen et al., 2010a). On the other hand, deficiency of *Cxcr4* specifically in cardiac myocytes did not affect heart function or remodeling after MI (Agarwal et al., 2010).

Together, these studies demonstrate a double-edged role of CXCR4 in the ischemic heart, and require further investigation of the role of CXCR4 and its chemokine ligands in the inflammatory processes associated with MI. In this context, also the alternative CXCR4 ligand MIF is upregulated in myocardium and plasma after MI (Yu et al., 2001, 2003) and can exert cardioprotection. Underlying mechanisms include activation of AMP-activated protein kinase (AMPK) (Miller et al., 2008), reduction of oxidative stress (Koga et al., 2011) or inhibition of *c*-Jun N-terminal kinase (JNK)-mediated apoptosis (Qi et al., 2009). An important role for the chemokine receptor CXCR2 on resident cardiac cells in MIF-mediated myocardial protection after ischemic injury was recently shown (Liehn et al., 2013a), but it remains unclear whether also MIF/CXCR4 signaling in cardiomyocytes may contribute to cardioprotection. Furthermore, MIF-induced recruitment and differentiation of protective EPCs through CXCR4 and CXCR2 may be involved in the cardioprotective effects of MIF (Simons et al., 2011; Asare et al., 2013; Kanzler et al., 2013). On the other hand, adverse effects of MIF through myocardial infiltration of inflammatory cells were revealed after prolonged ischemic injury in both MI and MI/IRI (Gao et al., 2011; White et al., 2013). Although these studies did not examine which MIF receptor was involved, a recent report demonstrated an important role for CXCR2 in mediating MIF-triggered monocyte recruitment in the ischemic heart (Liehn et al., 2013a). But also here, an additional involvement of MIF/CXCR4 interaction in ischemic inflammatory cell recruitment remains unclear. In conclusion, recent data indicate that similarly as CXCR4, MIF plays a double-edged role in myocardial ischemia. However, the relative importance of CXCR4 vs. other MIF receptors as CXCR2, in MIF-mediated effects remain unclear.

### CXCR4 in arterial injury-induced restenosis

The CXCL12/CXCR4 axis has been revealed to contribute to injury-induced restenosis, which is a major problem after coronary revascularization. CXCL12 expression is increased after vascular injury through enhanced hypoxia-inducible factor (HIF) 1α expression (Schober et al., 2003; Karshovska et al., 2007) and is preceded and mediated by apoptosis in the injured vessel wall (Zernecke et al., 2005). Systemic treatment of mice with a CXCL12 blocking antibody or a CXCR4 antagonist reduced injury-induced neointimal size and content of smooth muscle cells (SMCs), which are a driving force of neointimal hyperplasia. Similar results were obtained after transplantation with *Cxcr4*^−/−^ bone marrow or local treatment with a dysfunctional CXCL12 mutant (Schober et al., 2003; Zernecke et al., 2005; Karshovska et al., 2008; Hamesch et al., 2012). In these studies reduced SMC content was associated with a reduction in injury-induced mobilization of Lin^−^Sca1^+^ progenitor cells, which were shown to be incorporated into neointimal lesions and capable to differentiate into SMCs (Schober et al., 2003; Zernecke et al., 2005). This corresponded with previous observations that bone marrow-derived cells can be recruited to mechanically injured arteries, where they can differentiate into vascular SMCs (VSMCs) and even endothelial cells (ECs) (Sata et al., 2002; Tanaka and Sata, 2008).

Multiple studies have linked EPCs with enhanced reendothelialization and reduced neointimal hyperplasia after vessel injury (Werner et al., 2003; Kong et al., 2004). CXCR4 was shown to contribute to adhesion of *in vitro* mononuclear cell-derived EPCs to injured arteries, although to a lesser extent than CXCR2 (Hristov et al., 2007). Whereas CXCR4 blockade interfered with the capacity of infused EPCs to promote reendothelialization and reduce neointimal lesion size after carotid artery injury (Yin et al., 2010; Li et al., 2011), overexpression of CXCR4 promoted CXCL12-triggered migration and adhesion of EPCs *in vitro* and enhanced their capacity to promote endothelial recovery after vascular denudation *in vivo* (Chen et al., 2010b). However, it remains debated whether EPCs really affect injury-induced restenosis through direct incorporation in the injured vascular wall, or rather through paracrine effects on resident vascular cells by secreting mitogenic cytokines and growth factors as VEGF (Yoshioka et al., 2006; Iwata et al., 2010; Nemenoff et al., 2011; Hagensen et al., 2012; Merkulova-Rainon et al., 2012).

Furthermore, blockade of CXCR4 was shown to reduce cellular proliferation and the macrophage content of neointimal lesions after femoral artery injury, which was associated with reduced neointimal lesion size (Olive et al., 2008). In the same mouse model, ability of macrophage colony stimulating factor (M-CSF or CSF1) to accelerate injury-induced neointimal hyperplasia was abolished upon treatment with AMD3100. This was associated with reduced lesional incorporation of CXCR4^+^ cells despite enhanced white blood cell count in the peripheral blood, suggesting a detrimental role for CXCR4 in injury-induced neointimal hyperplasia in mediating the recruitment of inflammatory cells into neointimal lesions (Shiba et al., 2007).

In conclusion, blocking the CXCL12/CXCR4 axis interferes with injury-induced neointimal hyperplasia through reduced recruitment of CXCR4^+^ smooth muscle progenitors and inflammatory cells to the site of injury. On the other hand, CXCR4 enhances the ability of infused EPCs to adhere to injured vessels and promote reendothelialization. Although these data derive from *in vitro* cultured EPCs, which may behave different than the rather vaguely defined “circulating EPCs” (Steinmetz et al., 2010; Rennert et al., 2012), the current findings suggest a double-edged role for CXCR4 in injury-induced restenosis through recruitment of (progenitor) cells that either stimulate or interfere with neointimal hyperplasia. Such double-edged function was recently also observed upon tamoxifen-induced endothelial-specific deficiency of *Cxcr4* (*Cxcr4^EC−KO^*)in *apolipoprotein E*-deficient (*Apoe*^−/−^) mice, which significantly decreased mobilization of both circulating Sca1^+^Flk1^+^Cd31^+^ cells, often referred to as EPCs, and of Lin^−^Sca1^+^ cells upon wire-mediated injury of the carotid artery. Furthermore, *Cxcr4^EC−KO^ Apoe*^−/−^ mice showed a reduced reendothelialization efficiency, which was linked with a decrease in endothelial wound healing and *in vivo* proliferation. As a net result, endothelial-specific *Cxcr4* deficiency triggered the formation of larger neointimal lesions, displaying an increase in inflammatory macrophages but a reduced SMC content (Noels et al., 2014). Whether CXCR4 signaling also affects specific functions of VSMCs or macrophages in context of vascular injury remains to be investigated.

### CXCR4 in native atherosclerosis: progenitor cell mobilization

#### Vascular progenitor cells

In contrast to conditions of MI and injury-induced restenosis, not much is known about a potential involvement of bone marrow-derived vascular progenitor cells in native atherosclerosis, as has been recently summarized for EPCs (Du et al., 2012) and vascular smooth muscle progenitor cells (SPCs) (Merkulova-Rainon et al., 2012). It was shown that infusion of EPCs as well as treatment with AMD3100, which triggered EPC mobilization, enhanced plaque regression after normalization of plasma lipid levels in *Reversa* mice (Yao et al., 2012). In contrast, a study in *Apoe*^−/−^ mice did not reveal an atheroprotective effect of systemic AMD3100 treatment, but rather found AMD3100 to abolish beneficial effects of apoptotic body treatment on atherosclerosis. Endothelial apoptotic bodies were shown to contain miRNA126, which is transferred to neighboring ECs to induce the expression and release of CXCL12 by unleashing autoregulatory CXCR4 signaling. Injection of EC-derived apoptotic bodies into *Apoe*^−/−^ mice increased CXCL12 expression in atherosclerotic lesions and promoted progenitor cell mobilization and their recruitment to the endothelial lining of the lesions. Lesions of mice treated with apoptotic bodies were generally smaller in size and exhibited a less inflammatory phenotype with reduced macrophage and apoptotic cell content (Zernecke et al., 2009). Thus, despite contradictory findings on the effect of AMD3100 treatment, both studies suggest an atheroprotective function for CXCL12/CXCR4 signaling through mobilization of protective EPCs. Interestingly, it was revealed that patients with CAD show lower levels and a decreased migratory response of circulating EPCs (Vasa et al., 2001). In addition, systemic treatment of mice with CXCL12 in a partial ligation model—which induces advanced atherosclerotic lesions with an unstable phenotype—enhanced the recruitment of Lin^−^Sca1^+^ SPCs and promoted a more stable plaque phenotype (Akhtar et al., 2013). Such plaque-stabilizing role for SPCs was also suggested earlier by Zoll et al, who showed that injection of SPCs reduced atherosclerotic lesion size and improved lesion stability (Zoll et al., 2008). Together, these studies reveal an atheroprotective function for CXCL12/CXCR4 signaling through recruitment of protective EPCs and plaque-stabilizing SPCs.

However, others reported on an atheroprogressive role for vascular progenitor cells. Inducing apoptosis of rare lesional bone marrow-derived SMCs substantially decreased plaque size (Yu et al., 2011a), and George et al. found EPC transfer to increase atherosclerosis in mice (George et al., 2005). Contradictory findings on the presence or function of vascular progenitor cells in chronic atherosclerosis may be related to the atherosclerosis model, lesion stage, but also on the vague definition of such progenitor cells. Further research is definitively needed to improve phenotypical and functional characterization of vascular progenitor cell subsets in context of atherosclerosis before a role of the CXCR4/CXCL12 axis in their mobilization and potential functions in this pathology can be investigated in detail.

#### Hematopoietic progenitor cells

Interestingly, MI was recently revealed to accelerate atherosclerosis in mice. MI reduced CXCL12 expression in bone marrow through sympathetic nervous system activity and signaling through β 3 adrenergic receptor (β 3AR or ADRB3). In this way, MI enhanced mobilization of HSPCs from bone marrow niches and their hosting in the spleen, triggering myelopoiesis and increased atherosclerosis up to 3 months after coronary ligation (Dutta et al., 2012). Although the latter study did not address the underlying mechanisms of CXCL12 upregulation upon β 3AR blocking nor examine potential effects on CXCR4 expression or function, LaRocca *et al*. revealed a direct (physical) interaction of β 2 adrenergic receptors with CXCR4 resulting in the modification of the contractile nature of cardiomyocytes (Larocca et al., 2010). It is further known that β 2- and β 3 adrenergic receptors cooperate during progenitor cell mobilization with partial functional redundancy under stress (Mendez-Ferrer et al., 2010). Hence, one may speculate that Cxcr4 may also functionally interact with other adrenergic receptors, like β 3AR, in a direct or indirect way.

### CXCR4 in native atherosclerosis: cell type-specific functions?

In addition to a role for CXCR4 through progenitor cell mobilization, CXCR4 may affect native atherogenesis by modifying atherosclerosis-relevant cellular functions. CXCR4 expression has been described on many cell types including monocytes and macrophages, neutrophils (Bruhl et al., 2003), T-cells (Murphy et al., 2000), B-cells (Nie et al., 2004), mature ECs (Gupta et al., 1998), and SMCs (Nemenoff et al., 2008; Jie et al., 2010). All of these cells play distinct roles in the pathophysiology of atherosclerosis, but not much is known about the precise role of CXCR4 in individual cellular responses.

#### Monocytes and macrophages

Monocytes and macrophages have been proven to be of outstanding importance in the progression and development of mature atherosclerotic lesions and their depletion has been recognized atheroprotective already 20 years ago (Ylitalo et al., 1994). As the picture grows it becomes more and more evident that depletion of individual cell subsets does not serve as a realistic therapeutic approach, hence understanding the details of cell–cell interactions and communication gains importance (Weber and Noels, 2011).

#### CXCR4 expression and potential functions: studies of human cells

*Macrophages and foam cells.* CXCR4 is expressed on all monocyte subsets, with highest expression on classical human monocytes. This contrasts with observations in mice, which show highest CXCR4 levels on non-classical monocytes (Ingersoll et al., 2010), as will be discussed later in more detail. Gupta et al. revealed high expression of CXCR4 on human blood monocytes, which declined while they differentiated into macrophages, but restored again after 24 h, peaking at 7 days. Interestingly, CXCR4 expression on macrophages could be further upregulated by stimulation with oxidized low-density lipoprotein (oxLDL). From here the authors conclude that, although there is no direct evidence, restoration of CXCR4 expression on lesional macrophages and its further up-regulation by oxLDL during foam cell formation may contribute to migration of intimal foam cells and the subsequent progression of plaque growth (Gupta et al., 1999). Furthermore, CXCL12/CXCR4 signaling was linked with enhanced macropinocytosis in leukocytes (Tanaka et al., 2012), suggesting that a lack of CXCR4 may also influence (modified) lipid accumulation in macrophages and other lesional cells. In contrast, a recent study found CXCL12 to induce phagocytosis and the uptake of acetylated LDL in THP1-derived macrophages specifically through binding CXCR7 but not CXCR4 (Ma et al., 2013). In this context, the CXCR7 agonist CCX771 was recently shown to increase the uptake of very low-density LDL (VLDL) in adipocytes. Correspondingly, treatment of *Apoe*^−/−^ mice with CCX771 reduced the levels of circulating VLDL and decreased atherosclerosis (Li et al., 2014). Whether similar mechanisms can be identified in other cell types as macrophages remains to be examined, as are the exact mechanisms underlying CXCR7-mediated uptake of VLDL or modified lipids.

*Patients with CAD.* In patients with stable and unstable angina pectoris CXCR4 surface expression on peripheral blood mononuclear cells (PBMCs) was decreased and CXCL12 levels in patients with unstable angina pectoris were explicitly low. However, *in vitro* treatment of PBMCs from these patients with high concentrations of CXCL12 reduced mRNA and protein levels of chemokine ligands CCL2 and CXCL8, MMP9 and tissue factor, while increasing tissue inhibitor of metalloproteinases (TIMP) 1. Therefore, high (local) concentrations of CXCL12 may mediate anti-inflammatory and matrix-stabilizing effects promoting plaque stabilization and may be beneficial in angina pectoris and ACSs (Damas et al., 2002). Another study showed autocrine CXCL12 signaling to downregulate expression of runt-related transcription factor (RUNX) 3 in human monocytes/macrophages, thereby promoting a pro-angiogenic, but immunosuppressive phenotype of these cells (Sanchez-Martin et al., 2011).

*Compounds regulating CXCR4.* Several compounds were identified to modify CXCR4-mediated immune responses *in vitro*. Glucocorticoids have been demonstrated to upregulate CXCR4 expression on human blood monocytes and Caulfied et al. assume that increased CXCR4 expression sensitizes monocytes to tissue resident CXCL12, guiding monocytes away from sites of inflammation with supposingly lesser local CXCL12 release (Caulfield et al., 2002). The latter most likely contradicts another study, which revealed high expression of CXCL12 in SMCs, ECs and macrophages in human atherosclerotic plaques but not in normal vessels (Abi-Younes et al., 2000). This would rather argue for a chemotactic gradient of CXCL12 toward the site of inflammation, although this might be disease- and cell type-dependent.

As a second example, monocyte CXCR4 expression has been shown to be modulated by hydrogen sulfide (H_2_S) donors. H_2_S donors have lately been recognized as vasoprotective agents and changes in H_2_S may affect atherosclerosis. Interestingly, a synthetic slow H_2_S releaser (GYY4137) inhibited oxLDL-induced foam cell formation and cholesterol esterification in RAW264 cells and primary human monocytes, which was accompanied by decreased CXCR4 expression (Liu et al., 2013). In contrast, angiotensin-converting enzyme (ACE) inhibitors, widely used to treat high blood pressure by interfering with the renin-angiotensin system, did not affect CXCR4 expression on primary human monocytes and THP1 cells (Apostolakis et al., 2007). The same group also assessed if angiotensin I and II treatment would have a direct impact on chemokine receptor expression on THP1 cells, again CXCR4 expression was not altered (Apostolakis et al., 2010).

Conjugated linoleic acids (CLA) were shown to influence human peripheral blood monocyte function by suppressing CD18 expression, thereby reducing the number of β 2-integrins expressed on the external surface and decreasing adhesion to activated ECs. In addition, CLA reduced CXCR4 expression, resulting in an only minor initiation of “inside out” signaling. As a result, partial, but incomplete, activation of β 2-integrins further reduces adherence of leukocytes and their migration to CXCL12 (De Gaetano et al., 2013).

Statins, which lower intracellular cholesterol synthesis, are the *gold standard* to treat hyperlipidemia-associated atherosclerosis, but have also been reported to cause numerous other pleiotropic effects. To examine if statin withdrawal would affect human monocyte subsets in patients with stable CAD, statin treatment was cut off for 2 weeks. Subsequent evaluation of blood monocyte subsets did not reveal any differences in numbers, but downregulation of Toll-like receptor (TLR) 4 on all subsets and decreased expression of CXCR4 on classical monocytes (CD14^++^ CD16^−^) (Jaipersad et al., 2013). Interestingly, high doses of statin treatment were also shown to reduce general CXCL12 plasma levels in hyperlipidemic patients (Camnitz et al., 2012). However, it remains elusive whether statin treatment and a subsequent increase in CXCR4 expression, but decreased CXCL12 titers, point at a direct pro- or anti-atherosclerotic role of the CXCL12/CXCR4 axis.

Another mechanism, recently recognized to drive atherosclerotic lesion growth, is hypoxia (Marsch et al., 2013). Notably, hypoxia-induced upregulation of the transcriptional activator HIF1 triggers CXCR4 mRNA and protein expression in human monocytes. In addition, these cells showed increased migration toward CXCL12 under hypoxic conditions. Based on these findings the authors conclude that the hypoxia–HIF1–CXCR4 pathway may regulate cell trafficking and localization into hypoxic tissues, such as atherosclerotic lesions (Schioppa et al., 2003).

In contrast, CXCR4 expression on macrophages was suppressed in the presence of M-CSF. Increased M-CSF titers have been implicated in the pathogenesis of atherosclerosis and it was shown that M-CSF delivers a pro-atherogenic signal to human macrophages by stimulation of cholesterol accumulation and pro-inflammatory chemokine secretion. Thus, M-CSF-induced suppression of macrophage CXCR4 expression may point at an atheroprotective function of downstream CXCR4 signaling events (Irvine et al., 2009).

***CXCR4 expression and potential functions: mouse studies*.** The above-mentioned studies on human monocytes and macrophages suggest diverse potential roles for CXCR4 in atherosclerosis, still mouse studies are equally elusive, without a clear indication for a pro- or anti-atherogenic role for CXCL12/CXCR4. In contrast to human monocytes, CXCR4 in mouse is higher expressed on non-classical monocytes and it is not clear if this does necessarily imply functional differences in individual mouse and human monocyte subsets or not (Ingersoll et al., 2010).

One study reported an interesting finding using a mutant non-heparin sulfate-binding CXCL12 (HSmCXCL12). *In vitro*, HSmCXCL12 failed to promote transendothelial migration of PBMCs if used as chemoattractant in the bottom well of transwell plates, and inhibited the haptotactic response to wild-type CCL7, CXCL12, and CXCL8. Further, intravenous administration of HSmCXCL12 into mice also repressed the recruitment of lymphocytes and mononuclear phagocytes to air pouches injected with CXCL12. Moreover, repetitive administration of HSmCXCL12 *in vivo* reduced leukocyte-surface expression of CXCR4, and CXCL12-induced chemotaxis and adhesion. From here the authors conclude that non-heparin sulfate-binding variants of CXCL12 can mediate a powerful anti-inflammatory effect through induction of chronic CXCR4 internalization on leukocytes *in vivo*. Subsequently, this leads to receptor desensitization putatively explaining the functional deficits of these leukocytes (O'boyle et al., 2009). Hence, it could be interesting to carefully dissect the differential functional consequences of receptor desensitization through receptor internalization compared to CXCR4 blocking with AMD as reported by Zernecke et al. (2008).

A potential pro-inflammatory role for wild-type CXCL12 may also be deduced from findings from Liu et al., who revealed decreased CXCR4 expression on RAW264 cells and primary human monocytes treated with the H_2_S releaser GYY4137, which was introduced above. Administration of GYY4137 into *Apoe*^−/−^ mice receiving a high-fat diet for 4 weeks decreased atherosclerotic plaque formation and partially restored aortic endothelium-dependent relaxation. Further, intercellular adhesion molecule (ICAM) 1, TNF-α and interleukin (IL) 6 mRNA expression as well as superoxide generation in the aorta declined in mice treated with GYY4137 (Liu et al., 2013). Similarly, and again paralleling *in vitro* studies with human monocytes, CLA treatment of *Apoe*^−/−^ mice with already pre-established atherosclerosis induced lesion regression by reducing leukocyte adhesion and decreasing CD18 expression on classical monocytes (De Gaetano et al., 2013).

The above studies may point at a pro-atherogenic role of CXCR4 signaling in atherosclerosis; however this may strongly depend on the binding partner interacting with CXCR4. As already described, CXCL12 is not the only ligand for CXCR4 and MIF, an alternative ligand for CXCR4, has a strong pro-atherogenic impact. In this context, Bernhagen et al. revealed that antibody mediated-neutralization of MIF, but not CXCL12, induced atherosclerotic lesion regression in *Apoe*^−/−^ mice (Bernhagen et al., 2007). In line, *knock-out* of *Mif* in *Ldlr*^−/−^ mice did also result in diminished atherosclerosis (Pan et al., 2004). This suggests potential different roles of CXCR4 and its ligand CXCL12 in atherosclerosis through interplay of CXCR4 with MIF. Similarly, interplay of CXCL12 with other signaling molecules may modify its inflammatory effects. For example, hetero-complexes of high mobility group box (HMGB) 1 and CXCL12 were reported to induce inflammatory cell recruitment to injured tissue, which was not the case for each compound alone. Further, these complexes exclusively bound to CXCR4 inducing a conformational rearrangement of CXCR4, which differed from the single binding of CXCL12 to its receptor (Schiraldi et al., 2012).

#### Neutrophils

Treatment of mice with the CXCR4 antagonist AMD3100 induced cell egress from the bone marrow (Schiraldi et al., 2012), which is in line with findings by Zernecke et al. who described increased leukocytosis, mostly neutrophil mobilization, and enhanced lesion formation in *Apoe*^−/−^ receiving a cholesterol-rich diet for 12 weeks while supplemented with AMD. Interestingly, monocyte numbers were only moderately enhanced in these mice and, according to the authors, lesion growth was mainly attributable to increased plaque neutrophils and enhanced apoptosis (Zernecke et al., 2008).

Notably, a growing body of evidence underlines the role of neutrophils in atherogenesis (Drechsler et al., 2010, 2011; Doring et al., 2012) and it was recognized that the CXCL12/CXCR4 axis maintains neutrophil homeostasis primarily by regulation of neutrophil release from the bone marrow in a cell-autonomous fashion (Eash et al., 2009). It was further implicated that senescent neutrophils in the periphery expressing high levels of CXCR4 home back to the bone marrow to be cleared (Martin et al., 2003). In contrast, activated neutrophils downregulate CXCR4 expression putatively postponing their clearance (Bruhl et al., 2003; Martin et al., 2003).

#### Lymphocytes

CXCR4 on lymphocytes plays an essential role during B-cell development (Nagasawa et al., 1996) and T-cell homeostasis (Bleul et al., 1996b; Zou et al., 1998). Furthermore, the CXCL12/CXCR4 axis drives chemotaxis or fugetaxis of T-cells in various pathophysiological settings (Poznansky et al., 2000; Dunussi-Joannopoulos et al., 2002; Fernandis et al., 2003; Okabe et al., 2005; Zhang et al., 2005). Similarly, CXCL12 is able to trigger B-cell chemotaxis *in vitro* through CXCR4 (Klasen et al., 2014).

It became evident that the impact of B- and T-cells in atherosclerosis is strongly subset-dependent. While e.g., Th1 responses are known to be pro-atherosclerotic, regulatory T-cells haven been proven to be protective. Similarly, B1- and B2-cells exhibit diverse functions in lesion development (Weber and Noels, 2011). Nevertheless, studies dissecting the role of CXCR4 on T- and B-cells in the context of atherosclerosis are scarce. Two independent groups showed that lysophosphatidylcholine (LPC), a main phospholipid component of oxLDL, upregulates CXCR4 expression on Jurkat cells and human blood CD4^+^ T-cells. Further, the chemotactic ability of CD4^+^ T-cells toward CXCL12 and their production of pro-inflammatory cytokines were increased in the presence of LPC. Hence, the ill alliance of LPC and CXCL12 in atherosclerotic lesions may amplify pro-inflammatory responses by stimulation of CD4^+^ T-cells and subsequent plaque growth (Han et al., 2004; Hara et al., 2008). Interestingly, it was also shown that excess of mineralocorticoids, mainly aldosterone, drive CAD by cardiac and renal fibrosis, as well as hypertension. Here, Chu et al. imply a specific role of the CXCL12/CXCR4 axis in the detrimental consequences of mineralocorticoid excess and render CXCL12 explicitly responsible for the accumulation of T-cells in fibrotic tissue (Chu et al., 2011). From patients with abdominal aortic aneurysm (AAA) we further learn that T- and B-cells recruited to sites of AAA express high levels of CXCR4 and exhibit a pro-inflammatory signature. Hence, CXCR4/CXCL12 interactions may strongly impact on the recruitment and retention of inflammatory lymphocytes infiltrating AAAs (Ocana et al., 2008). In contrast, acute stress induced by public speaking did not enhance the number of CXCR4 expressing T-cells, but increased the frequency of T-cells expressing CXCR2, CXCR3, and CCR5. Therefore, cardiac sympathetic activation may lead to EC and T-cell activation subsequently driving acute flooding of atherosclerotic lesions with pro-inflammatory mediators (Bosch et al., 2003).

In addition to CXCL12-triggered effects, CXCR4 is able to mediate MIF-induced B-cell chemotaxis, and T-cell chemotaxis and arrest *in vitro* (Bernhagen et al., 2007; Klasen et al., 2014). Blockade of MIF in *Apoe*^−/−^ mice on high-fat diet resulted in the formation of smaller atherosclerotic lesions displaying a reduced macrophage and T-cell content, supporting a role for MIF in T-cell chemotaxis also in the context of atherosclerosis (Bernhagen et al., 2007).

#### Platelets

CXCR4 expression (mRNA, protein) was reported on platelets (Wang et al., 1998; Kowalska et al., 1999) and although platelets lack nuclei and many organelles and are mainly known for their important role in blood coagulation, their impact on immunological and inflammatory responses, in particular atherosclerosis, should not be underestimated (Lievens and Von Hundelshausen, 2011). Addition of CXCL12 to platelets from healthy donors induced platelet aggregation, which could be inhibited by blocking CXCR4. The latter implies an atherogenic, pro-thrombotic, and plaque-destabilizing role for the CXCL12/CXCR4 axis *in vivo* (Falk et al., 1995; Abi-Younes et al., 2000). In contrast, others report CXCL12 to be a weak platelet agonist, however still amplifying platelet activation, adhesion and chemokine release triggered by low doses of primary platelet agonists, such as adenosine diphosphate (ADP) and thrombin, or arterial flow conditions (Kowalska et al., 1999; Gear et al., 2001). Furthermore, CXCL12 gradients could induce platelet migration and transmigration *in vitro* involving PI3K signaling (Kraemer et al., 2010). In addition, recent work showed CXCL12 to trigger CXCR4 internalization and cyclophilin A-dependent CXCR7 externalization on (mouse and human) platelets, resulting in prolonged platelet survival. Mice lacking the cytosolic chaperone cyclophilin A showed less CXCL12-induced rescue of platelets from activation-induced apoptosis through CXCR7 engagement. Hence, differential regulation of CXCR4/CXCR7 surface expression on platelets upon CXCL12 exposure at sites of platelet activation/accumulation may orchestrate platelet survival, subsequently impacting on platelet-mediated physiological mechanisms (Chatterjee et al., 2014).

#### Vascular endothelial cells

***CXCR4 expression in arterial ECs*.** Expression of CXCR4 on various types of vascular ECs has been widely reported (Hillyer et al., 2003), however, it should be emphasized that ECs are a very heterogeneous population, with ECs from different anatomic sites differing in basal gene expression, localization and function (Aird, 2007). Further, one has to carefully distinguish between expression on venous and arterial ECs (Dela Paz and D'amore, 2009). Unfortunately, many studies extrapolate e.g., *in vitro* findings generated with human umbilical vein endothelial cells (HUVECs) to arteriosclerosis, which is at least daring.

In a study examining CXCR4 expression following vessel wall injury in porcine coronary arteries, CXCR4 expression could be shown 24 h to 7 days after injury, but only in lymphocytes, granulocytes and myelo-fibroblasts entering the injured tissue (Jabs et al., 2007). However, others investigated CXCR4 expression in human carotid artery specimens, where CXCR4 was abundantly expressed by lesional ECs and only marginally in minimally diseased endothelium (Molino et al., 2000; Melchionna et al., 2005). Similarly, Gupta et al. showed *CXCR4* mRNA expression in human coronary artery ECs, although it is not clear if these cells originated from inflamed or steady-state endothelium (Gupta et al., 1998). CXCR4 was also shown to be expressed (mRNA and protein) by cultured bovine aortic ECs (BAECs), in cryo-sections of rabbit thoracic aortas (Volin et al., 1998) and in mouse aortic endothelium (Melchionna et al., 2010). For BAECs it was further demonstrated that resting BAECs accumulate CXCR4 protein in cytoplasmic granules, while migrating BAECs display a diffuse surface expression of CXCR4 (Feil and Augustin, 1998).

In addition, *in vitro* studies with human aortic ECs (HAECs) revealed enhanced CXCR4 surface expression and CXCL12-induced chemotaxis in the presence of VEGF or basic fibroblast growth factor (bFGF) 48 h after stimulation. Notably, interferon (IFN)-γ, lipopolysaccharide (LPS) or CXCL12 did not elevate the surface expression of CXCR4 (Salcedo et al., 1999).

***CXCR4 expression in venous and microvascular ECs*.** Upregulation of CXCR4 surface expression after addition of VEGF and bFGF was also seen in human microvascular ECs and HUVECs (Salcedo et al., 1999, 2003). In contrast, Schutyser et al. do not report changes in *CXCR4* mRNA expression in human microvascular ECs after treatment with VEGF, but describe augmented CXCR4 expression (mRNA and protein) after serum starvation and/or hypoxic treatment of microvascular ECs (Schutyser et al., 2007). Further, hypoxia is also an important regulator of the CXCL12/CXCR4 axis in HUVECs by enhancing CXCR4 expression (Ceradini et al., 2004; Jin et al., 2012). In general hypoxia does cause lowering of local pH, and pH changes are also known to occur upon physical exercise and hemodynamic shear stress, as well as in pathological states including cardiac ischemia. In this context Melchionna et al. reported that acidosis decreased CXCR4 surface expression on mouse aortic ECs *in vivo* and on HUVECs *in vitro* in a HIF1α-dependent manner (Melchionna et al., 2010).

Notably, mouse microvascular ECs were also shown to augment CXCR4 expression *in vitro* in response to erythromycin, an anti-inflammatory antibiotic drug used for treatment of chronic inflammatory diseases. The authors assume that beneficial effects of erythromycin are partly due to CXCR4-expressing ECs recruited to sites of tissue injury (Takagi et al., 2009). However, since microvascular ECs also comprise a broad variety of ECs, for example of dermal, brain, heart or pancreatic islets origin, it remains elusive how any differential regulation of CXCR4 expression described above would impact on atherosclerotic plaque development.

***Role of CXCR4 in ECs?*** Concerning possibly relevant functions of endothelial CXCR4 signaling in context of atherosclerosis, several putatively athero-relevant findings were described in HUVECs, but confirmations in arterial ECs are pending. For example, laminar shear stress suppresses CXCR4 expression in HUVECs while low shear stress favors CXCR4 expression, subsequently resulting in increased EC apoptosis and CCL2 and CXCL8 release (Melchionna et al., 2005).

Another study revealed enhanced release of CXCL12 by HUVECs after oxLDL treatment and a subsequently increased migratory and adhesive response of MSCs (Li et al., 2010). It was further demonstrated that MIF facilitates leukocyte rolling on stimulated HUVECs while siRNA-mediated *knock-down* of endothelial MIF resulted in decreased expression of E-selectin, ICAM-1, vascular cell adhesion molecule (VCAM) 1, CXCL8, and CCL2 (Cheng et al., 2010).

In addition, it seems that not only VEGF may regulate CXCR4 expression, but it was also shown that CXCL12 treatment increased VEGF protein expression in human microvascular ECs after serum starvation (Saxena et al., 2008), underlining the role of CXCL12 in angiogenesis. Given the CXCL12/CXCR4 axis to be angiogenic in general (Ara et al., 2005; Unoki et al., 2010), but also in tumor development (Domanska et al., 2013) and potentially in atherosclerotic lesions (Di Stefano et al., 2009), and angiogenesis being considered to increase plaque vulnerability, the question still remains if this reflects an important unfavorable role of CXCR4 in atherosclerosis. In a different approach, blocking of TLR2 resulted in increased angiogenic capacity of HUVECs, putatively mediated via association of TLR2 with CXCR4 and subsequently enhanced CXCR4 signaling. Consequently, *knock-down* of CXCR4 in the presence of TLR2 blocking antibodies revealed less angiogenesis. From these data the authors conclude that TLR2 blocking might serve as a promising therapeutic approach in e.g., MI or peripheral artery disease by promoting revascularization. However, considering angiogenesis pro-atherogenic, TLR2 blocking might have detrimental consequences in the context of plaque stability and development, as already mentioned above (Wagner et al., 2013). Nevertheless, it was also shown that the inflammatory mediators IFN-γ and TNF-α decrease CXCR4 and CXCL12 expression in HUVECs, thereby decreasing their angiogenic capacity (Gupta et al., 1998; Salvucci et al., 2004). Interestingly, the latter contradict findings from Salcedo et al, who showed no difference in CXCR4 expression in HAECs after IFN-γ treatment (Salcedo et al., 1999). Yet, it should be emphasized again that HUVECs and HAECs might exert totally different responses in the presence of the same stimulus, underlining again the importance of caution in generalizing findings from ECs of different origin.

#### Vascular smooth muscle cells

VSMCs are highly specialized cells controlling contraction and regulation of blood vessel diameter, blood pressure, and blood flow. Moreover, VSMCs play a critical role in secretion of extracellular matrix components, which determine the mechanical properties of mature blood vessels. Differentiated VSMCs in adult blood vessels proliferate at very low rates and retain high plasticity, which enables changes in phenotype, referred to as phenotypic switching (Owens et al., 2004). Phenotypic switching of VSMCs is considered an important pathophysiological mechanism in atherosclerosis (Gomez and Owens, 2012).

***CXCR4 expression in vascular SMCs*.** Not much is known about the expression and function of CXCR4 in mature VSMCs. Several studies reported no CXCR4 expression on human or bovine (aortic) SMCs (Gupta et al., 1998; Volin et al., 1998). In contrast, Schecter et al. were the first to claim a functional CXCR4 expression on human aortic SMCs, as the addition of CXCL12 or envelope proteins of HIV specifically binding CXCR4 did induce tissue factor activity in human aortic SMCs (Schecter et al., 2001). Similarly, it was demonstrated that HIV can infect arterial (lesional) human SMCs and blocking CXCR4 in human aortic SMCs *in vitro* did significantly reduce their viral load. Again, direct expression of CXCR4 on aortic SMCs was not investigated. Yet, the authors still claim that HIV infection of VSMCs through CXCR4 may be one reason why HIV patients are more susceptible to develop atherosclerosis (Eugenin et al., 2008). Nevertheless, Li et al. revealed CXCR4 protein expression on human saphenous vein SMCs (Li et al., 2009) and others later reported CXCR4 expression (RNA, protein) on mouse medial SMCs (Nemenoff et al., 2008), rat aortic SMCs (Jie et al., 2010; Pan et al., 2012) and human aortic SMCs (Weber et al., unpublished data).

***Role of CXCR4 in vascular SMCs?*** As mentioned before, phenotypic switching of VSMCs from a contractile to a synthetic secretory phenotype and their assumed migration from the medial to the intimal arterial wall, where they secrete pro-inflammatory mediators, is considered a pathophysiological mechanism in atherogenesis (Gomez and Owens, 2012). On the other hand, intimal SMCs do also stabilize atherosclerotic lesions by fibrous cap formation. This contrasts the pathology of injury-induced restenosis and neointimal hyperplasia, which are mainly driven by SMC proliferation. Hence, the contribution of SMCs to lesion formation is strongly context-dependent.

In a rat model of diabetes, a metabolic disorder associated with a higher prevalence of atherosclerosis, high glucose levels were shown to trigger activation, proliferation, and enhanced chemotaxis of VSMCs via stimulation of the CXCL12/CXCR4 axis (Jie et al., 2010). Correspondingly, salvianolic acid B (*Salvia miltiorrhiza)*, used to treat cardiovascular diseases in traditional Chinese medicine, was shown to inhibit CXCL12/CXCR4-mediated proliferation, migration and subsequently neointimal hyperplasia by VSMCs in a balloon angioplasty-induced neointima formation model in rats. Here salvianolic acid B directly decreased surface expression of CXCR4 on aortic rat SMCs (Pan et al., 2012).

Further, lesion reduction in *Mif^−/−^Apoe*^−/−^ mice was attributed to a reduction in lesional SMC proliferation, cysteine protease expression, and elastinolytic and collagenolytic activities (Pan et al., 2004). Notably, oxLDL, supposed to be an important trigger of atherogenesis and plaque growth, was shown to induce rat aortic SMC proliferation. This effect could even be further enhanced by addition of CXCL12 and came along with diminished SMC apoptosis. It remains open if this effect would be beneficial through plaque stabilization, or detrimental because of intimal hyperplasia, as discussed above (Li et al., 2013). As mentioned before, vein graft failure after bypass grafting is a major problem and may be associated with neointimal hyperplasia and accelerated atherosclerosis. In this context, Zhang et al. demonstrate that CXCL12/CXCR4 signaling might be a crucial step in vein graft atherosclerosis and contribute to SMC-mediated vein graft neointimal hyperplasia in mice. Furthermore, CXCR4-mediated recruitment of inflammatory (progenitor) cells to the vein graft may add to this picture (Zhang et al., 2012).

Also, it was described that CXCL12 stimulates pro-MMP2 expression in human aortic SMCs via CXCR4 in association with the epidermal growth factor receptor *in vitro*. The authors conclude that CXCR4 expands its signaling repertoire by cross-talking with other receptors, pointing at an important role of ligands engaged in receptor cross-talk as critical players in CAD (Kodali et al., 2006).

## Genome-wide association studies reveal CXCL12 as an important candidate gene in CAD

Genome-wide association studies of European ancestry revealed 2 single nucleotide polymorphisms (SNPs) on locus 10q11.21, 80 kB downstream of *CXCL12*, to be significantly associated with CAD and MI (Burton et al., 2007; Samani et al., 2007; Kathiresan et al., 2009; Farouk et al., 2010; Schunkert et al., 2011), although genome-wide significance was not reached in 2 other studies (Ripatti et al., 2010; Peden et al., 2011) (Box 4). Whether and how the risk alleles of these SNPs rs1746048(C/C) and rs501120(T/T) affect expression level and/or function of the CXCL12 protein is currently still unclear.

A significant association was revealed between the CAD risk genotype for rs501120 (T/T) and reduced CXCL12 plasma levels (Kiechl et al., 2010). Likewise, patients with angina displayed reduced CXCL12 plasma levels compared to healthy controls. The reduction was even higher in case of unstable disease (Damas et al., 2002), suggesting an atheroprotective role for CXCL12. Remarkably, patients with angina showed a significant reduction in CXCR4 surface expression on PBMCs, despite increased levels of *CXCR4* RNA transcripts, but its connection to disease remains unclear (Damas et al., 2002).

In contrast, a recent study revealed the risk alleles of these SNPs to be associated with higher CXCL12 plasma levels, rather suggesting a pro-atherogenic role for CXCL12 (Mehta et al., 2011). Additional large-scale studies in human patients with CAD/MI investigating CXCL12 plasma levels in relation to SNPs and disease would be helpful to get better insight into the role of CXCL12 in this pathology. Furthermore, animal studies are definitively required to unravel disease-associated functions in a cell-specific and molecular way.

Also for the alternative CXCR4 ligand MIF, SNPs have been identified that are associated with cardiovascular disease, as recently summarized (Tillmann et al., 2013). Although the effect of these SNPs on MIF expression or function remain unknown, enhanced MIF plasma levels in patients with ACS (Muller et al., 2012) and the identification of a high MIF plasma level as a risk factor for adverse coronary events in CAD patients with impaired glucose tolerance or type 2 diabetes mellitus (Makino et al., 2010) may support a pro-inflammatory role of plasma MIF in CAD.

## Clinical perspectives and conclusion

**Figure 3:**
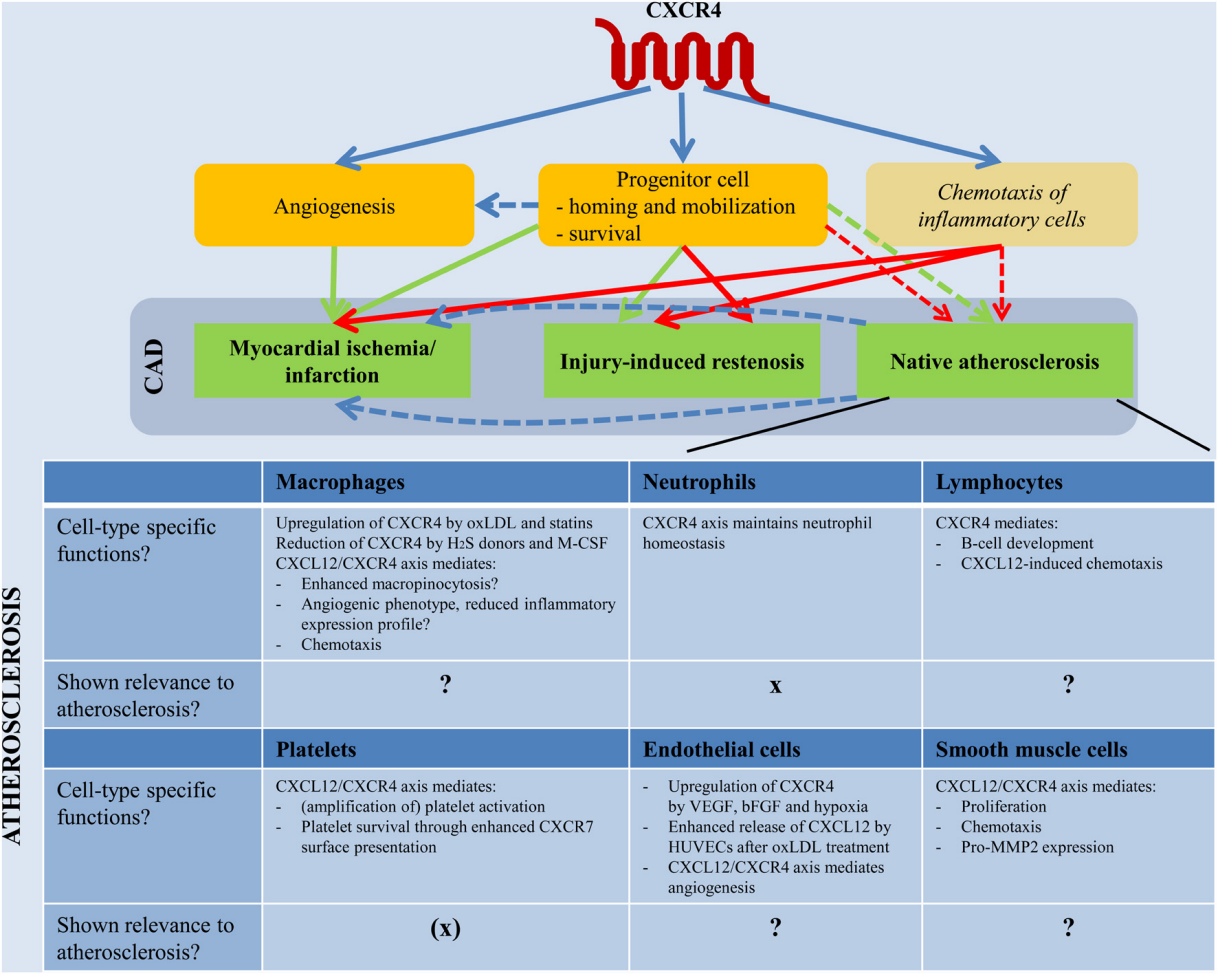
**Involvement of CXCR4 in CAD.** The chemokine receptor CXCR4 plays a role in angiogenesis. Furthermore, it is an important regulator of homing, mobilization and survival of progenitor cells. This has linked CXCR4 with a role in myocardial ischemia and injury-induced restenosis, but its significance in the context of native atherosclerosis remains unclear. CXCR4 has also been reported to mediate leukocyte chemotaxis in specific inflammatory diseases. A similar role in inflammatory cell recruitment has been suggested in the context of myocardial ischemia, but the importance of CXCR4-induced leukocyte recruitment to atherosclerotic lesions *in vivo* remains to be further addressed. The current view mainly emphasizes the involvement of inflammatory chemokines instead of the homeostatic chemokine CXCL12 in mediating atherogenic leukocyte recruitment. However, CXCR4 can mediate both CXCL12- and MIF-induced chemotaxis of B- and T-cells *in vitro*, and is also expressed on a subset of monocytes, requiring further research of its function in atherogenic leukocyte recruitment *in vivo*. Also, it remains unclear which cell type-specific functions of CXCR4 may be important in context of atherosclerosis, with currently only scarce information on potential cellular functions in most cell types present in atherosclerotic lesions. For more details, we refer to the text. Green arrows indicate beneficial effects, red arrows indicate detrimental effects. The interrelation between different pathologies belonging to CAD is visualized. The lower panels indicate relevance of CXCR4-involving cell type-specific functions to atherosclerotic plaque formation. bFGF, basic fibroblast growth factor; CAD, coronary artery disease; H2S, hydrogen sulfide; M-CSF, macrophage colony stimulating factor; MMP, matrix metallopeptidase; oxLDL, oxidized low-density lipoprotein; VEGF, vascular endothelial growth factor.

In conclusion, the role of CXCR4 in native atherosclerosis remains elusive, with only few isolated studies shedding some light on the effect of CXCL12/CXCR4 signaling on cell type-specific functions involved in inflammation or atherosclerosis. In contrast, the CXCL12/CXCR4 axis has been better explored in context of injury-induced restenosis and myocardial ischemia, in which a role for this chemokine ligand/receptor axis has mostly been linked to the mobilization and recruitment of progenitor cells and, to a lesser extent, inflammatory cells (Figure 3). The CXCR4 antagonist AMD3100, also known as Plerixafor, has been approved as mobilizer of hematopoietic stem cells in combination with G-CSF in treatment of patients with non-Hodgkin's lymphoma and multiple myeloma, and many other small molecule inhibitors of CXCR4 are in clinical trial or under investigation (Debnath et al., 2013). However, a potential future application of such inhibitors in treatment of patients with CAD is currently only speculative. Although mobilization of progenitor cells has been associated with cardioprotection in context of myocardial ischemia and also initial clinical trials for stem cell therapy after MI are encouraging, many important aspects of such therapy—as optimal cell type, dose, time and method of administration, long-term effects—remain to be investigated (Sanganalmath and Bolli, 2013). Furthermore, contrasting reports on the effect of AMD3100 treatment on cardiac outcome after MI warns for a further evaluation of underlying mechanisms, and also the recently revealed double-edged role of CXCR4 in myocardial ischemia necessitates a careful evaluation of drugs interfering with CXCL12/CXCR4 signaling. In addition, possible unwanted side effects need to be cautiously monitored. For example, one patient study examining the effects of progenitor cell mobilization on cardiac function after MI was terminated early due to enhanced in-stent restenosis (Kang et al., 2004). Also, a closer investigation of the effect of CXCR4 antagonists on progenitor cell mobilization in context of different cardiovascular disease settings or upon different dosage or administration methods seems interesting, as contrasting findings were reported on the effect of continuous administration of the CXCR4 antagonist AMD3465 on the mobilization of Lin^−^Sca1^+^ cells in context of native atherosclerosis *vs* injury-induced restenosis (Karshovska et al., 2008; Zernecke et al., 2008).

Furthermore, additional studies are required to unravel in more detail the cellular processes in which CXCR4 is involved and the underlying molecular mechanisms. In this context, complexness of CXCR4-associated biological and mechanistic aspects is significantly being increased by the identification of MIF as a secondary chemokine ligand for CXCR4, and of CXCR7 as an alternative receptor for CXCL12. Intertwining of chemokine receptor signaling may enhance fine tuning and optimization of leukocyte chemotaxis in physiological conditions. In addition, it may increase the possibilities for designing therapeutics interfering with only selective aspects of chemokine signaling, for example by targeting chemokine receptor heterodimerization (Koenen and Weber, 2010). But again, this necessitates a better understanding of biological and mechanistic aspects of all involved chemokine ligand/receptor axes and their interplay.

### Conflict of interest statement

Christian Weber is consultant of Carolus Therapeutics Inc., a US-based biotech company developing chemokine-based anti-inflammatory strategies. The authors declare that the research was conducted in the absence of any commercial or financial relationships that could be construed as a potential conflict of interest.
