# Supplementary material for: The CXCL12/CXCR4 chemokine ligand/receptor axis in cardiovascular disease
Source: Front Physiol. 2014 Jun 11;5:212. doi: 10.3389/fphys.2014.00212 (PMC4052746; doi:10.3389/fphys.2014.00212)
Supplement: Supplementary file 1 [file DataSheet1.PDF]

Supplemental Table 1. Cell type-specific expression of CXCL12 and CXCR4.

**CXCL12 expression upregulated/downregulated by**				
Stimulus	***Species***	***Cell type***	***Up/down***	***Reference***
	**Human**			
General inflammation		atherosclerotic plaque	up	(Abi-Younes et al., 2000)
Statins		plasma	down	(Camnitz et al., 2012)
Angina pectoris		plasma	down	(Damas et al., 2002)
oxLDL		HUVECs	up	(Li et al., 2010)
IFNγ, TNFα		HUVECs	down	(Gupta et al., 1998;Salvucci et al., 2004)
Apoptotic bodies		HUVECs	up	(Zernecke et al., 2009)
	**Mouse**			
Apoptotic bodies		aortic ECs	up	(Zernecke et al., 2009)
**CXCR4 expression upregulated/downregulated by**				
Stimulus	***Species***	***Cell type***	***Up/down***	***Reference***
	**Human**	**Monocytes/Macrophages**		
None		classical monocytes	high	(Ingersoll et al., 2010)
None		non-classical monocytes	present	(Ingersoll et al., 2010)
oxLDL		macrophages	up	(Gupta et al., 1999)
Glucocorticoids		blood monocytes	up	(Caulfield et al., 2002)
H_2S releaser		RAW264<br>blood monocytes	up	(Liu et al., 2013)
ACE-inhibitors		THP1<br>blood monocytes	no change	(Apostolakis et al., 2007)
Angiotensin I & II		THP1	no change	(Apostolakis et al., 2010)
Conjugated linoleic acid		blood monocytes	down	(de Gaetano et al., 2013)
Statins		classical monocytes	down	(Jaipersad et al., 2013)
Hypoxia		monocytes	up	(Schioppa et al., 2003)
Colony-stimulating factor 1		macrophages	down	(Irvine et al., 2009)
		**Neutrophils**		
None		blood neutrophils	present	(Martin et al., 2003)
IFNγ, TNFα		blood neutrophils	down	(Bruhl et al., 2003)
Time after isolation		blood neutrophils	up	(Bruhl et al., 2003)
		**Lymphocytes**		
None		B- and T-cells	present	(Bleul et al., 1996;Nagasawa et al., 1996;Zou et al., 1998)
Lysophosphatidylcholin		Jurkat<br>blood $CD4^+$ T-cells	up	(Han et al., 2004;Hara et al., 2008)
Abdominal aortic aneurysm		T- and B-cells	high	(Ocana et al., 2008)
‘Public speaking’ (acute stress)		T-cells	down	(Bosch et al., 2003)
		**Platelets**		
none		blood	present	(Wang et al., 1998;Kowalska et al., 1999)
CXCL12		blood	(up)	(Abi-Younes et al., 2000)

Stimulus	*Species*	*Cell type*	*Up/down*	*Reference*
		**Leukocytes in general**		
Angina pectoris		blood leukocytes	down	(Damas et al., 2002)
		**Vascular endothelial cells**		
Endarterectomy specimens		lesional ECs	present	(Molino et al., 2000;Melchionna et al., 2005)
Not clear		coronary artery ECs	present	(Gupta et al., 1998)
VEGF, bFGF		aortic ECs	up	(Salcedo et al., 1999)
IFNγ, LPS, CXCL12		aortic ECs	no change	(Salcedo et al., 1999)
VEGF, bFGF		microvascular ECs, HUVECs	up	(Salcedo et al., 1999;Salcedo et al., 2003)
VEGF		microvascular ECs	no change	(Schutyser et al., 2007)
Serum starvation, hypoxia		microvascular ECs	up	(Schutyser et al., 2007)
Acidosis		HUVECs	down	(Melchionna et al., 2010)
Laminar shear stress		HUVECs	down	(Melchionna et al., 2005)
Low shear stress		HUVECs	up	(Melchionna et al., 2005)
IFNγ, TNFα		HUVECs	down	(Gupta et al., 1998;Salvucci et al., 2004)
		**Vascular smooth muscle cells**		
None		aortic SMCs	not present	(Gupta et al., 1998;Volin et al., 1998)
CXCL12		aortic SMCs	(up?) no direct proof	(Schecter et al., 2001)
HIV		arterial SMCs	(up?) no direct proof	(Eugenin et al., 2008)
Cyclic stretch		saphenous vein SMCs	up	(Li et al., 2009)
	**Mouse**	**Monocytes/Macrophages**		
None		classical monocytes	present	(Ingersoll et al., 2010)
None		non-classical monocytes	high	(Ingersoll et al., 2010)
		**Lymphocytes**		
Aldosterone		T-cells	up	(Chu et al., 2011)
		**Neutrophils**		
None		blood / bone marrow	present	(Martin et al., 2003)
		**Leukocytes in general**		
Non-heparin sulfate binding CXCL12		leukocytes	down	(O'Boyle et al., 2009)
		**Vascular endothelial cells**		
Erythromycin		microvascular ECs	up	(Takagi et al., 2009)
None		aortic ECs	present	(Melchionna et al., 2010)
Acidosis		aortic ECs	down	(Melchionna et al., 2010)

Stimulus	***Species***	***Cell type***	***Up/down***	***Reference***
	**Others**			
Vessel wall injury	porcine	lymphocytes, granulocytes, myelo-fibroblasts	present	(Jabs et al., 2007)
	bovine	aortic ECs	present	(Volin et al., 1998)
	rabbit	thoracal aorta ECs	present	(Volin et al., 1998)
None	bovine	aortic SMCs	not present	(Volin et al., 1998)
Glucose	rat	aortic SMCs	up	(Jie et al., 2010)
Salvianolic acid B	rat	aortic SMCs	down	(Pan et al., 2012)

References

Abi-Younes, S., Sauty, A., Mach, F., Sukhova, G.K., Libby, P., and Luster, A.D. (2000). The stromal cell-derived factor-1 chemokine is a potent platelet agonist highly expressed in atherosclerotic plaques. *Circ Res* 86**,** 131-138.

Apostolakis, S., Krambovitis, E., Vlata, Z., Kochiadakis, G.E., Baritaki, S., and Spandidos, D.A. (2007). CX3CR1 receptor is up-regulated in monocytes of coronary artery diseased patients: impact of pre-inflammatory stimuli and renin-angiotensin system modulators. *Thrombosis research* 121**,** 387-395.

Apostolakis, S., Vlata, Z., Vogiatzi, K., Krambovitis, E., and Spandidos, D.A. (2010). Angiotensin II up-regulates CX3CR1 expression in THP-1 monocytes: impact on vascular inflammation and atherogenesis. *Journal of thrombosis and thrombolysis* 29**,** 443-448.

Bleul, C.C., Fuhlbrigge, R.C., Casasnovas, J.M., Aiuti, A., and Springer, T.A. (1996). A highly efficacious lymphocyte chemoattractant, stromal cell-derived factor 1 (SDF-1). *The Journal of experimental medicine* 184**,** 1101-1109.

Bosch, J.A., Berntson, G.G., Cacioppo, J.T., Dhabhar, F.S., and Marucha, P.T. (2003). Acute stress evokes selective mobilization of T cells that differ in chemokine receptor expression: a potential pathway linking immunologic reactivity to cardiovascular disease. *Brain, behavior, and immunity* 17**,** 251-259.

Bruhl, H., Cohen, C.D., Linder, S., Kretzler, M., Schlondorff, D., and Mack, M. (2003). Post-translational and cell type-specific regulation of CXCR4 expression by cytokines. *European journal of immunology* 33**,** 3028-3037.

Camnitz, W., Burdick, M.D., Strieter, R.M., Mehrad, B., and Keeley, E.C. (2012). Dose-dependent Effect of Statin Therapy on Circulating CXCL12 Levels in Patients with Hyperlipidemia. *Clinical and translational medicine* 1**,** 23.

Caulfield, J., Fernandez, M., Snetkov, V., Lee, T., and Hawrylowicz, C. (2002). CXCR4 expression on monocytes is up-regulated by dexamethasone and is modulated by autologous CD3+ T cells. *Immunology* 105**,** 155-162.

Chu, P.Y., Zatta, A., Kiriazis, H., Chin-Dusting, J., Du, X.J., Marshall, T., and Kaye, D.M. (2011). CXCR4 antagonism attenuates the cardiorenal consequences of mineralocorticoid excess. *Circulation. Heart failure* 4**,** 651-658.

Damas, J.K., Waehre, T., Yndestad, A., Ueland, T., Muller, F., Eiken, H.G., Holm, A.M., Halvorsen, B., Froland, S.S., Gullestad, L., and Aukrust, P. (2002). Stromal cell-derived factor-1alpha in unstable angina: potential antiinflammatory and matrix-stabilizing effects. *Circulation* 106**,** 36-42.

De Gaetano, M., Dempsey, E., Marcone, S., James, W.G., and Belton, O. (2013). Conjugated linoleic acid targets beta2 integrin expression to suppress monocyte adhesion. *Journal of immunology* 191**,** 4326-4336.

Eugenin, E.A., Morgello, S., Klotman, M.E., Mosoian, A., Lento, P.A., Berman, J.W., and Schecter, A.D. (2008). Human immunodeficiency virus (HIV) infects human arterial smooth muscle cells in vivo and in vitro: implications for the pathogenesis of HIV-mediated vascular disease. *The American journal of pathology* 172**,** 1100-1111.

Gupta, S.K., Lysko, P.G., Pillarisetti, K., Ohlstein, E., and Stadel, J.M. (1998). Chemokine receptors in human endothelial cells. Functional expression of CXCR4 and its transcriptional regulation by inflammatory cytokines. *J Biol Chem* 273**,** 4282-4287.

Gupta, S.K., Pillarisetti, K., and Lysko, P.G. (1999). Modulation of CXCR4 expression and SDF-1alpha functional activity during differentiation of human monocytes and macrophages. *Journal of leukocyte biology* 66**,** 135-143.

Han, K.H., Hong, K.H., Ko, J., Rhee, K.S., Hong, M.K., Kim, J.J., Kim, Y.H., and Park, S.J. (2004). Lysophosphatidylcholine up-regulates CXCR4 chemokine receptor expression in human CD4 T cells. *Journal of leukocyte biology* 76**,** 195-202.

Hara, Y., Kusumi, Y., Mitsumata, M., Li, X.K., and Fujino, M. (2008). Lysophosphatidylcholine upregulates LOX-1, chemokine receptors, and activation-related transcription factors in human T-cell line Jurkat. *Journal of thrombosis and thrombolysis* 26**,** 113-118.

Ingersoll, M.A., Spanbroek, R., Lottaz, C., Gautier, E.L., Frankenberger, M., Hoffmann, R., Lang, R., Haniffa, M., Collin, M., Tacke, F., Habenicht, A.J., Ziegler-Heitbrock, L., and Randolph, G.J. (2010). Comparison of gene expression profiles between human and mouse monocyte subsets. *Blood* 115**,** e10-19.

Irvine, K.M., Andrews, M.R., Fernandez-Rojo, M.A., Schroder, K., Burns, C.J., Su, S., Wilks, A.F., Parton, R.G., Hume, D.A., and Sweet, M.J. (2009). Colony-stimulating factor-1 (CSF-1) delivers a proatherogenic signal to human macrophages. *Journal of leukocyte biology* 85**,** 278-288.

Jabs, A., Okamoto, E., Vinten-Johansen, J., Bauriedel, G., and Wilcox, J.N. (2007). Sequential patterns of chemokine- and chemokine receptor-synthesis following vessel wall injury in porcine coronary arteries. *Atherosclerosis* 192**,** 75-84.

Jaipersad, A.S., Shantsila, E., Blann, A., and Lip, G.Y. (2013). The effect of statin therapy withdrawal on monocyte subsets. *European journal of clinical investigation* 43**,** 1307-1313.

Jie, W., Wang, X., Zhang, Y., Guo, J., Kuang, D., Zhu, P., Wang, G., and Ao, Q. (2010). SDF-1alpha/CXCR4 axis is involved in glucose-potentiated proliferation and chemotaxis in rat vascular smooth muscle cells. *International journal of experimental pathology* 91**,** 436-444.

Kowalska, M.A., Ratajczak, J., Hoxie, J., Brass, L.F., Gewirtz, A., Poncz, M., and Ratajczak, M.Z. (1999). Megakaryocyte precursors, megakaryocytes and platelets express the HIV co-receptor CXCR4 on their surface: determination of response to stromal-derived factor-1 by megakaryocytes and platelets. *Br J Haematol* 104**,** 220-229.

Li, F., Guo, W.Y., Li, W.J., Zhang, D.X., Lv, A.L., Luan, R.H., Liu, B., and Wang, H.C. (2009). Cyclic stretch upregulates SDF-1alpha/CXCR4 axis in human saphenous vein smooth muscle cells. *Biochemical and biophysical research communications* 386**,** 247-251.

Li, M., Yu, J., Li, Y., Li, D., Yan, D., Qu, Z., and Ruan, Q. (2010). CXCR4 positive bone mesenchymal stem cells migrate to human endothelial cell stimulated by ox-LDL via SDF-1alpha/CXCR4 signaling axis. *Exp Mol Pathol* 88**,** 250-255.

Liu, Z., Han, Y., Li, L., Lu, H., Meng, G., Li, X., Shirhan, M., Peh, M.T., Xie, L., Zhou, S., Wang, X., Chen, Q., Dai, W., Tan, C.H., Pan, S., Moore, P.K., and Ji, Y. (2013). The hydrogen sulfide donor, GYY4137, exhibits anti-atherosclerotic activity in high fat fed apolipoprotein E(-/-) mice. *British journal of pharmacology* 169**,** 1795-1809.

Martin, C., Burdon, P.C., Bridger, G., Gutierrez-Ramos, J.C., Williams, T.J., and Rankin, S.M. (2003). Chemokines acting via CXCR2 and CXCR4 control the release of neutrophils from the bone marrow and their return following senescence. *Immunity* 19**,** 583-593.

Melchionna, R., Porcelli, D., Mangoni, A., Carlini, D., Liuzzo, G., Spinetti, G., Antonini, A., Capogrossi, M.C., and Napolitano, M. (2005). Laminar shear stress inhibits CXCR4 expression on endothelial cells: functional consequences for atherogenesis. *FASEB journal : official publication of the Federation of American Societies for Experimental Biology* 19**,** 629-631.

Melchionna, R., Romani, M., Ambrosino, V., D'arcangelo, D., Cencioni, C., Porcelli, D., Toietta, G., Truffa, S., Gaetano, C., Mangoni, A., Pozzoli, O., Cappuzzello, C., Capogrossi, M.C., and Napolitano, M. (2010). Role of HIF-1alpha in proton-mediated CXCR4 down-regulation in endothelial cells. *Cardiovascular research* 86**,** 293-301.

Molino, M., Woolkalis, M.J., Prevost, N., Pratico, D., Barnathan, E.S., Taraboletti, G., Haggarty, B.S., Hesselgesser, J., Horuk, R., Hoxie, J.A., and Brass, L.F. (2000). CXCR4 on human endothelial cells can serve as both a mediator of biological responses and as a receptor for HIV-2. *Biochim Biophys Acta* 1500**,** 227-240.

Nagasawa, T., Hirota, S., Tachibana, K., Takakura, N., Nishikawa, S., Kitamura, Y., Yoshida, N., Kikutani, H., and Kishimoto, T. (1996). Defects of B-cell lymphopoiesis and bone-marrow myelopoiesis in mice lacking the CXC chemokine PBSF/SDF-1. *Nature* 382**,** 635-638.

O'boyle, G., Mellor, P., Kirby, J.A., and Ali, S. (2009). Anti-inflammatory therapy by intravenous delivery of non-heparan sulfate-binding CXCL12. *FASEB journal : official publication of the Federation of American Societies for Experimental Biology* 23**,** 3906-3916.

Ocana, E., Perez-Requena, J., Bohorquez, J.C., Brieva, J.A., and Rodriguez, C. (2008). Chemokine receptor expression on infiltrating lymphocytes from abdominal aortic aneurysms: role of CXCR4-CXCL12 in lymphoid recruitment. *Atherosclerosis* 200**,** 264-270.

Pan, C.H., Chen, C.W., Sheu, M.J., and Wu, C.H. (2012). Salvianolic acid B inhibits SDF-1alpha-stimulated cell proliferation and migration of vascular smooth muscle cells by suppressing CXCR4 receptor. *Vascular pharmacology* 56**,** 98-105.

Salcedo, R., Wasserman, K., Young, H.A., Grimm, M.C., Howard, O.M., Anver, M.R., Kleinman, H.K., Murphy, W.J., and Oppenheim, J.J. (1999). Vascular endothelial growth factor and basic fibroblast growth factor induce expression of CXCR4 on human endothelial cells: In vivo neovascularization induced by stromal-derived factor-1alpha. *Am J Pathol* 154**,** 1125-1135.

Salcedo, R., Zhang, X., Young, H.A., Michael, N., Wasserman, K., Ma, W.H., Martins-Green, M., Murphy, W.J., and Oppenheim, J.J. (2003). Angiogenic effects of prostaglandin E2 are mediated by up-regulation of CXCR4 on human microvascular endothelial cells. *Blood* 102**,** 1966-1977.

Salvucci, O., Basik, M., Yao, L., Bianchi, R., and Tosato, G. (2004). Evidence for the involvement of SDF-1 and CXCR4 in the disruption of endothelial cell-branching morphogenesis and angiogenesis by TNF-alpha and IFN-gamma. *Journal of leukocyte biology* 76**,** 217-226.

Schecter, A.D., Berman, A.B., Yi, L., Mosoian, A., Mcmanus, C.M., Berman, J.W., Klotman, M.E., and Taubman, M.B. (2001). HIV envelope gp120 activates human arterial smooth muscle cells. *Proceedings of the National Academy of Sciences of the United States of America* 98**,** 10142-10147.

Schioppa, T., Uranchimeg, B., Saccani, A., Biswas, S.K., Doni, A., Rapisarda, A., Bernasconi, S., Saccani, S., Nebuloni, M., Vago, L., Mantovani, A., Melillo, G., and Sica, A. (2003). Regulation of the chemokine receptor CXCR4 by hypoxia. *J Exp Med* 198**,** 1391-1402.

Schutyser, E., Su, Y., Yu, Y., Gouwy, M., Zaja-Milatovic, S., Van Damme, J., and Richmond, A. (2007). Hypoxia enhances CXCR4 expression in human microvascular endothelial cells and human melanoma cells. *European cytokine network* 18**,** 59-70.

Takagi, Y., Hashimoto, N., Phan, S.H., Imaizumi, K., Matsuo, M., Nakashima, H., Hashimoto, I., Hayashi, Y., Kawabe, T., Shimokata, K., and Hasegawa, Y. (2009). Erythromycin-induced CXCR4 expression on microvascular endothelial cells. *American journal of physiology. Lung cellular and molecular physiology* 297**,** L420-431.

Volin, M.V., Joseph, L., Shockley, M.S., and Davies, P.F. (1998). Chemokine receptor CXCR4 expression in endothelium. *Biochemical and biophysical research communications* 242**,** 46-53.

Wang, J.F., Liu, Z.Y., and Groopman, J.E. (1998). The alpha-chemokine receptor CXCR4 is expressed on the megakaryocytic lineage from progenitor to platelets and modulates migration and adhesion. *Blood* 92**,** 756-764.

Zernecke, A., Bidzhekov, K., Noels, H., Shagdarsuren, E., Gan, L., Denecke, B., Hristov, M., Koppel, T., Jahantigh, M.N., Lutgens, E., Wang, S., Olson, E.N., Schober, A., and Weber, C. (2009). Delivery of MicroRNA-126 by Apoptotic Bodies Induces CXCL12-Dependent Vascular Protection. *Sci. Signal.* 2**,** ra81-.

Zou, Y.R., Kottmann, A.H., Kuroda, M., Taniuchi, I., and Littman, D.R. (1998). Function of the chemokine receptor CXCR4 in haematopoiesis and in cerebellar development. *Nature* 393**,** 595-599.
